# Metabolomic profile in pancreatic cancer patients: a consensus-based approach to identify highly discriminating metabolites

**DOI:** 10.18632/oncotarget.6808

**Published:** 2016-01-01

**Authors:** Iole Maria Di Gangi, Tommaso Mazza, Andrea Fontana, Massimiliano Copetti, Caterina Fusilli, Antonio Ippolito, Fulvio Mattivi, Anna Latiano, Angelo Andriulli, Urska Vrhovsek, Valerio Pazienza

**Affiliations:** ^1^ Department of Food Quality and Nutrition, Research and Innovation Centre, Fondazione Edmund Mach (FEM), San Michele all'Adige, TN, Italy; ^2^ Unit of Bioinformatics, I.R.C.C.S. “Casa Sollievo della Sofferenza” Hospital, San Giovanni Rotondo, FG, Italy; ^3^ Unit of Biostatistics I.R.C.C.S. “Casa Sollievo della Sofferenza” Hospital, San Giovanni Rotondo, FG, Italy; ^4^ Gastroenterology Unit, I.R.C.C.S. “Casa Sollievo della Sofferenza” Hospital, San Giovanni Rotondo, FG, Italy

**Keywords:** metabolomic, pancreatic cancer

## Abstract

**Purpose:**

pancreatic adenocarcinoma is the fourth leading cause of cancer related deaths due to its aggressive behavior and poor clinical outcome. There is a considerable variability in the frequency of serum tumor markers in cancer' patients. We performed a metabolomics screening in patients diagnosed with pancreatic cancer.

**Experimental Design:**

Two targeted metabolomic assays were conducted on 40 serum samples of patients diagnosed with pancreatic cancer and 40 healthy controls. Multivariate methods and classification trees were performed.

**Materials and Methods:**

Sparse partial least squares discriminant analysis (SPLS-DA) was used to reduce the high dimensionality of a pancreatic cancer metabolomic dataset, differentiating between pancreatic cancer (PC) patients and healthy subjects. Using Random Forest analysis palmitic acid, 1,2-dioleoyl-sn-glycero-3-phospho-rac-glycerol, lanosterol, lignoceric acid, 1-monooleoyl-rac-glycerol, cholesterol 5α,6α epoxide, erucic acid and taurolithocholic acid (T-LCA), oleoyl-L-carnitine, oleanolic acid were identified among 206 metabolites as highly discriminating between disease states. Comparison between Receiver Operating Characteristic (ROC) curves for palmitic acid and CA 19-9 showed that the area under the ROC curve (AUC) of palmitic acid (AUC=1.000; 95% confidence interval) is significantly higher than CA 19-9 (AUC=0.963; 95% confidence interval: 0.896-1.000).

**Conclusion:**

Mass spectrometry-based metabolomic profiling of sera from pancreatic cancer patients and normal subjects showed significant alterations in the profiles of the metabolome of PC patients as compared to controls. These findings offer an information-rich matrix for discovering novel candidate biomarkers with diagnostic or prognostic potentials.

## INTRODUCTION

Pancreatic cancer (PC) is the fourth leading cause of cancer related deaths in the western world [1]. Due to the absence of early symptoms, the lack of effective systemic therapies and its aggressiveness, pancreatic cancer is characterized by a poor prognosis with only less than 5% of PC patients surviving up to 5 years [2].

The high morbidity and mortality associated with PC determine an urgent need for diagnostic biomarkers, as best approach to alert for this malignancy. Nowadays, the carbohydrate antigen CA 19-9 represents the most useful biochemical marker to distinguish PC patients from non-cancerous conditions, and to monitor PC recurrence following surgery [3]. However, different studies have been performed in the attempt to discover and improve minimally invasive and early diagnostic tools for PC, and several alternative tissues, serum and stool potential biomarkers have been proposed [4]. Tissue markers include genetic alterations of KRAS, p16/CDKN2A, TP53, SMAD4, that, in association with RNF43 and GNAS genes mutations, occur with high frequency also in PC precursor lesions [5, 6]. Alternative serum biomarkers emerged during the last decades, such as inhibitory cytokine-1 (MIC-1), CEACAM1, osteopontin (OPN), MUC-1, HSP70 and ULBP2 [7–12], and panels of other emerging serum markers, capable of discriminating PC patients from healthy subjects, have been also reported [7]. Similarly, a number of evidences highlighted alteration of specific microRNAs expression as promising blood- and tissue-based biomarkers for PC [13], and the aberrant methylation of BMP3 gene was suggested as a stool marker for PC patients compared to healthy controls [14].

Recently scientists are taking advantage of metabolomics approaches in order to have a complete picture of biological cells, tissue and organs to try to find new biomarkers [15]. As altered metabolite profiles are associated with disease, metabolomics approach represent an additional opportunity and a powerful tool to detect perturbations in the metabolome. In this study we performed a metabolomic screening in patients diagnosed with pancreatic cancer and in healthy controls.

## RESULTS

### Inter-class diversity assessment of metabolite profiles

**Figure 1:**
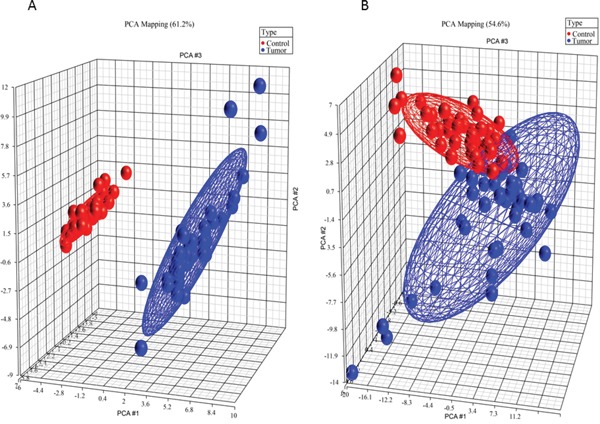
Principal component analysis 3D plots of LIPIDS (left) and metabolic profile (right) data sets 3D representation of patients, grouped by disease state. Blue dots are diseased patients, while red dots are healthy patients. The first three components are represented by the axes of the plot and capture the most variance of the data.

Inter-class diversity was assessed by principal component analysis. Figure 1A (different classes of lipids) shows how ellipsoids grouping samples that belong to same classes are far away from each other, thereby indicating a net diversity between classes. Metabolite profiles analyzed by the biocrates kit were quite of minor differentiating ability between groups (Figure 1B), still lying on orthogonal semi axes. However, this was indicative of the presence of a differential set of metabolites between diseased and healthy subjects.

### Consensus-based metabolites selection

**Figure 2:**
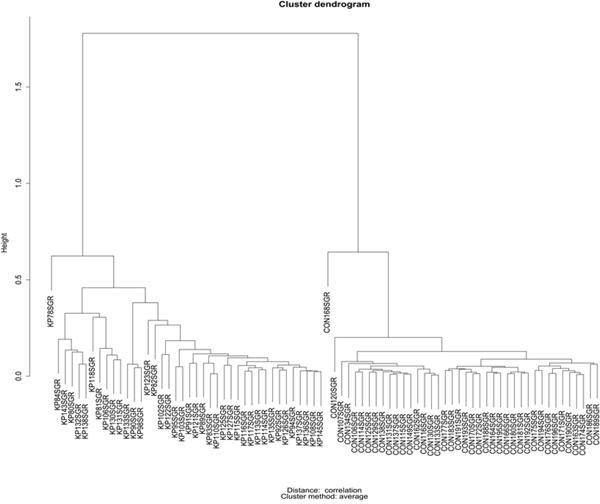
Hierarchical clustering for the 16 selected metabolites listed in Table 1

**Figure 3:**
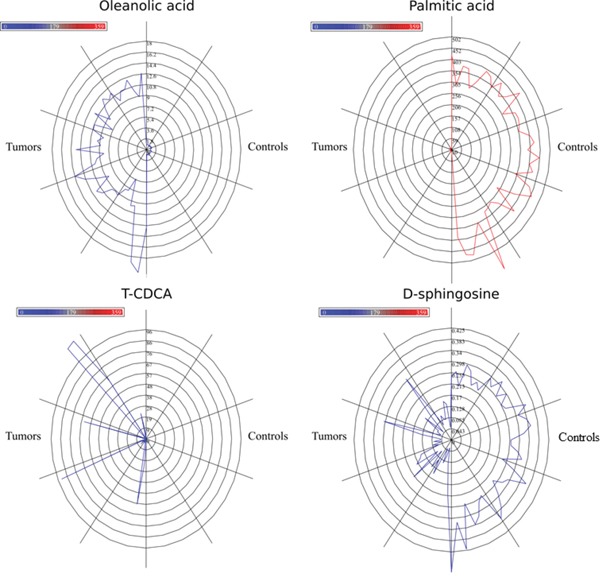
Star plots for four most discriminating metabolites Blue lines are related to tumor samples. Red lines refer to control samples. Radius increases with metabolite levels.

**Figure 4:**
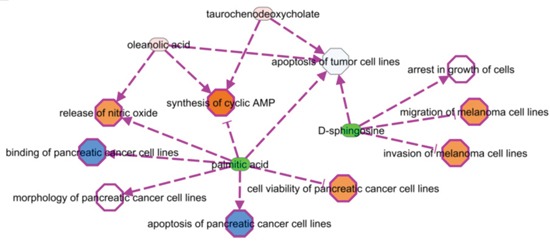
Metabolites involvement in critical functions and pathways related to the cancer onset/progression Green color means down-regulation, (light) red up-regulation of the four metabolites (small circles). Octagons represent relevant functions, which might be inhibited - if colored in blue - or activated - if colored in orange -, according to the up-regulation or down-regulation levels of the connected metabolites. Activation or inhibition states are inferred by literature evidences (PMIDs: 14688288, 24648512, 17440087, 12524422, 12810709).

We selected 16 metabolites, listed in Table 1, by intersecting the results of the three above-mentioned methods and then performed a hierarchical clustering (Figure 2). Four of these metabolites showed the highest cooperative discriminant potential, even though at a different extent: palmitic acid and oleanolic acid were the most perturbed metabolites. D-sphingosine and T-CDCA (taurochenodeoxycholate) were mildly discriminating factors. T-CDCA, in particular, was up-regulated in a few tumor patients (Figure 3). Metabolites were then subjected to functional enrichment analysis by Ingenuity Pathway Analysis. Over-represented functions were mostly correlated with pancreatic cancer onset/progression. As shown in Figure 4, palmitic acid and D-sphingosine modulate cell viability, apoptosis, cell cycle, migration and invasion in the frame of pancreatic cancer. Green color means down-regulation, red up-regulation of the four metabolites (small circles). Octagons represent relevant functions, which might be inhibited - if colored in blue - or activated - if colored in orange -, according to the up-regulation or down-regulation levels of the connected metabolites.

**Table 1:** List of 16 metabolites obtained by intersecting the results of sPLS-DA, Greedy stepwise and Genetic Search analyses

Metabolites	Which sPLS-DA Component
Lysophosphatidylcholine C16:0	1
Lysophosphatidylcholine C18:0	1
Phosphatidylcholine (32:1)	1
Cholesterol 5α,6α epoxide	2
1,2-dioleoyl-sn-glycero-3-phospho-rac-glycerol	1
D-sphingosine	1
Lanosterol	1
Erucic acid	1
Palmitic acid	1
Behenic acid	1 and 2
Lignoceric acid	1
Oleanolic acid	1
Glyceryltrilinoleate	1
1-monooleoyl-rac-glycerol	1 and 3
Glycolithocholic acid	-
Taurochenodeoxycholic acid	3

### Results from random forest: selection of the top discriminating metabolites

**Figure 5:**
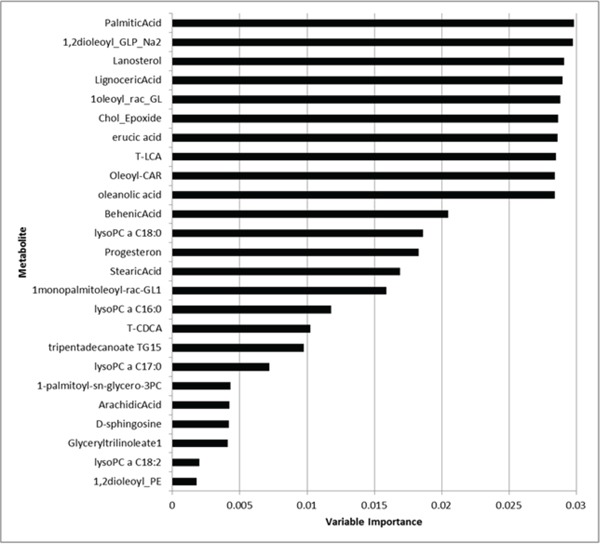
Variable importance values of the top 25 metabolites (from the most to the less important)

We run RF analysis by randomly selecting 20 metabolites as candidates for each node split of each tree included into the forest. The number of selected metabolites is the only user-required parameter of the RF and the choice of such metabolites randomly varies from tree to tree. By default, an acceptable value is represented by the square root of the number of metabolites. As the total number of metabolites was 206, we should select at least 14 metabolites and, indeed, we arbitrary decided to be more conservative and select 20 metabolites, which represented about 10% of the total number of metabolites. Figure 5 depicts the importance values of the top 25 metabolites (from the most to the less relevant). Medians of metabolite levels in patients with pancreatic cancer and in healthy donor were reported in Supplementary Table 1 whilst the full list of variable importance for all metabolites was further reported in Supplementary Table 2. We found that palmitic acid, 1,2-dioleoyl-sn-glycero-3-phospho-rac-glycerol, lanosterol, lignoceric acid, monooleoyl-rac-glycerol, cholesterol 5α,6α epoxide, erucic acid, T-LCA, oleoyl-L-carnitine, oleanolic acid clearly achieved the highest importance values than all other metabolites.

### Results from ROC curve analysis: discriminatory power of the top discriminating metabolites

The estimation of the AUC was performed for all metabolites and results were reported in Supplementary Table 3. As shown, for each metabolite, the best threshold (cut-off), at which sensitivity and specificity were jointly maximized was further reported. As shown, all metabolites previously found at the RF analysis (i.e., palmitic acid, 1,2-dioleoyl-sn-glycero-3-phospho-rac-glycerolcholesterol 5α,6α epoxidelanosterol, lignoceric acid, 1oleoyl_rac_GL, cholesterol_epoxide, erucic acid, T-LCA, oleoyl-L-carnitine, oleanolic acid) achieved the highest discriminatory power (AUC=1.00, 95%CI: 1.00-1.00), suggesting that all the estimated cut-offs points perfectly discriminate patients form healthy controls.

### CA19.9 vs palmitic acid as tumor discriminant marker

Tumor markers including carcinoembryonic antigen (CEA) or carbohydrate antigen 19-9 (CA19-9) are frequently determined at the time of diagnosis in patients with pancreatic cancer. To date, CA19-9 is the only biomarker routinely used, and FDA approved, for the clinical management of PC. Although several studies indicated a suboptimal prognostic relevance of this marker in pancreatic cancer, due to its low sensitivity and specificity [3], it has certain limitations in diagnosis and for screening purposes of the population at risk, which results in a difficult task to distinguish pancreatic cancer from benign tumors with normal CA19-9.

**Figure 6:**
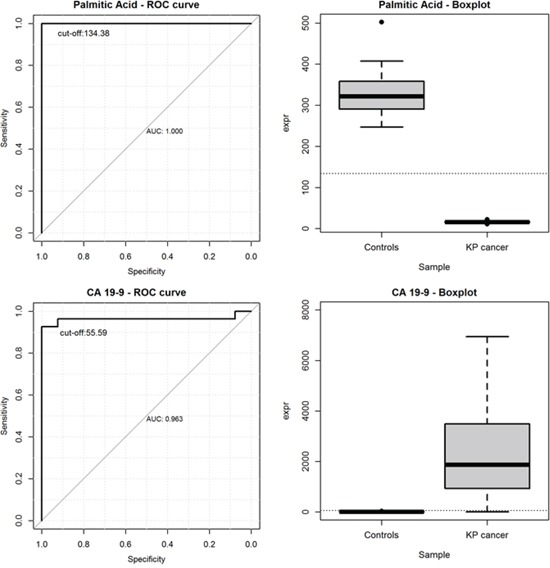
ROC curves and the boxplots of metabolite levels for palmitic acid (upper left and upper right) and for CA 19-9 (bottom left and bottom right)

Among all screened metabolites, we found that palmitic acid achieved the highest discriminatory power to distinguish PC patients from healthy controls. It was also used to investigate its prognostic ability in comparison to the baseline levels of CA 19-9. Plots of the ROC curves and the boxplots of metabolite levels were reported in Figure 6 for palmitic acid (upper left and upper right) and for CA 19-9 (bottom left and bottom right), separately. We found that the AUC for CA 19-9 was 0.963, along with a 95%CI of 0.896-1.000. The best threshold was found at the value of 55.59 μM, where CA 19-9 achieved a sensitivity of 92.6% and a specificity of 100%. Similarly, the AUC for palmitic acid was 1.000, along with a 95%CI of 1.000-1.000. The best cut-off was found at the value of 134.38 μM, where palmitic acid achieved a sensitivity of 100% and a specificity of 100%.

## DISCUSSION

Along with multiple genetic, epigenetic, and growth signaling alterations, cancer cells reprogram several metabolic pathways [16, 17]. Therefore in the cancer research field, metabolomics studies may lead to an enhanced understanding of disease mechanisms and to the discovery of new diagnostic biomarkers; they may also improve understanding of mechanisms for drug efficacy with the intent to tailor the therapeutic regimen to the individual patient.

Several reports suggested various potential biomarkers for pancreatic cancer in pancreatic tissue, such as leucine, isoleucine, valine, lactate, alanine, phosphocholine, glycerophosphocholine, taurine, and betaine [18]; taurine, lactate, creatine, and glutamate [19], in serum (3-hydroxybutyrate, 3-hydroxyisovalerate, lactate, trimethylamine-N-oxide, isoleucine, triglyceride, leucine, and creatine [20]; glutamate, acetone, 3-hydroxybutyrate, glucose, phenylalanine, formate, mannose, ethanol, asparagine, creatine, proline, and glycerol, plasma (N-methylalanine, lysine, glutamine, phenylalanine, arachidonic acid, lysophosphatidylcholine (18:2), phosphatidylcholine (34:2), phosphatidylethanolamine (26:0), tauroursodeoxycholic acid, taurocholic acid, deoxycholylglycine, and cholylglycine) [21], in urine (acetoacetate, citrate, creatinine, glucose, glycine, hippurate, 3-hydoxyisovalerate, leucine, 2-phenylacetamide, and trigonelline [15], and in saliva (leucine, isoleucine, tryptophan, valine, glutamate, phenylalanine, glutamine and aspartate) [22].

Perturbations of several metabolic pathways including glycolysis, amino acid metabolism, nucleotide and glycerophospholipid metabolism with increased fatty acid synthesis and changes in fatty acid metabolism have been proposed as determinants of the onset and progression of pancreatic cancer [23].

Free fatty acids, are relevant mediators of the proinflammatory milieu [24] and specifically the saturated free fatty acid palmitate, correlate positively with concentrations of the proinflammatory cytokine IL-6 [25].

Moreover, experimental studies have suggested that polyunsaturated fatty acids (PUFAs), but not monounsaturated (MUFAs) or saturated fatty acids (SFAs), inhibit human pancreatic cancer cell growth, which indicate the relationship between fats consumption and risk of pancreatic cancer may rely on the level of specific fatty acids intake [26, 27]. SFA promotes insulin resistance, whereas MUFA and PUFA improve insulin resistance [28], which might be involved in pancreatic cancer development [28, 29] such as in other types of cancer [30].

The excess of saturated free fatty acids, such as palmitic acid, that induces lipotoxicity in hepatocytes, has been implicated in the development of non-alcoholic fatty liver disease also associated with insulin resistance. In human and mouse hepatocytes palmitic acid, at a lipotoxic concentration, triggers early activation of endoplasmic reticulum (ER) stress-related kinases, inducing the apoptotic transcription factor CHOP, activating caspase 3 and increases the percentage of apoptotic cells. These effects concurred with decreased IR/IRS1/Akt insulin pathway [31]. In the present study, the concentrations of palmitic acid were 20 times lower in pancreatic cancer patients than healthy subjects, whereas oleanolic acid levels were 10 times higher in PC than control group (Supplementary Table 1). In addition by Ingenuity Pathway Analysis the same metabolites resulted as mostly perturbed with the highest discriminatory power (AUC=1.00, 95%CI: 1.00-1.00) between the patients and controls.

In addition, animal models have shown that saturated free fatty acids preferentially upregulate the production of proinflammatory cytokines (including TNF-α and IL-6) via mechanisms mediated in part through the Toll-like receptor 4/nuclear factorkB pathway [32, 33]. Furthermore, free fatty acids are an important source of reactive oxygen species through increased lipid peroxidation and correlate positively with increased plasma concentrations of the proinflammatory adipokine leptin [34].

In previous studies, human serum extracts enriched for selected hydroxylated polyunsaturated ultra long-chain fatty acids (GTAs) resulted to protect against inflammation through the down-regulation of NFκB and several pro-inflammatory markers in both human colon cancer and RAW264.3 mouse macrophage cells exposed to lipopolysaccharide [35]. GTA-treated cells also showed reduced proliferative capacity through a pro-apoptotic mechanism [35]. In colon cancer, for example, low-serum concentrations of GTA-446, an anti-inflammatory polyunsaturated long-chain fatty acid, was proposed by Ritchie and colleagues as a new risk factor [36]. Moreover GTAs act analogously to the resolvins and protectins [37], protecting the body against the accumulation of chronic inflammation over time. Compromised levels with age are suspected to favor the establishment of a pro-inflammatory environment, and ultimately lead to the DNA damage observed in many tumors.

Ritchie and colleagues [23], using a non-targeted metabolomics approach based on high-resolution, flow-injection Fourier transform ion cyclotron resonance mass spectrometry (FI-FTICR-MS), generated comprehensive metabolomic profiles from the serum of PC patients and disease-free subjects, isolating than the high discriminating ability of PC-594 metabolite between PC and healthy subjects. The latest recent findings by Ritchie and co-workers, who evaluated the potential clinical benefit of screening based on PC-594 to identify subjects with PC or a high-risk of developing PC, suggested that PC-594, belonging to the same metabolic system as GTA-446, could be involved in inflammatory processes of subjects with chronic pancreatitis and could be causally involved in the establishment of PC [36].

Lastly, palmitic acid is cytotoxic to β-cells in models of obesity and diabetes, as well as normal β-cells [38] and has been shown to induce apoptosis in exocrine pancreatic AR42J cells [39].

In the present study, the concentrations of palmitic acid were 20 times lower in pancreatic cancer patients than healthy subjects, whereas oleanolic acid levels were 10 times higher in PC than control group (Supplementary Table 5). In addition by Ingenuity Pathway Analysis the same metabolites resulted as mostly perturbed with the highest discriminatory power (AUC=1.00, 95%CI: 1.00-1.00) between the patients and controls.

The relative variable importance (VIMP) ranking values from RF analysis were for palmitic acid and oleanolic acid, respectively, of 100% and 95.3% (Supplementary Table 2), showing a clear predictive potential and a substantial larger importance of the two metabolites in respect to all others.

Oleanolic acid (OA) belongs to the triterpenoids family and is widely distributed in the skin and seeds of different edible fruits, including olives and white or red grapes, fruits and some medicinal herbs and it has been reported to have antioxidant, anti-inflammatory, antidiabetic, antimutagenicity and recently, anti-tumor activity for its marked effects and low toxicity [40].

The traditional Mediterranean diet, characterized by the consumption of foods such as grapes, wine, must, raisins, olives and virgin olive oil, has been associated with a low incidence of cancer [41]. Previously, it has been described that oleanolic acid and palmitic acid possess cardioprotective effects [42, 43], anti-inflammatory effects [44], and antiproliferative effects in highly invasive cancer cells and induce apoptosis in a wide variety of tumor cells including human prostate cancer cells [45], hepatocellular carcinoma cells [46], human pancreatic cells [47], liver cancer, lung cancer, osteosarcoma cells, human leukemia cells, human melanoma cells and colon cancer cells, among others [48].

OA in particular, could have potential chemopreventive activity in human breast cancer [49]: at low concentrations, it is a natural compound that acts as an antioxidant and prevents oxidative DNA damage in human mammary epithelial cells.

Recently, the anti-tumor activity of oleanolic acid has attracted much attention for its marked effects and low toxicity. OA can be also synthetized by humans as autodefense mechanisms. Several studies have shown that this metabolite could inhibit proliferation and induce apoptosis in a wide variety of tumor cells including colon cancer, breast cancer, liver cancer, lung cancer, osteosarcoma cells, human leukemia cells and human melanoma cells. Recently, Wei and colleagues have examined the anti-tumor activity of oleanolic acid on pancreatic cancer cells. The results of these studies provide evidence that it is able to arrest the cell cycle and induce apoptosis, possibly via ROS-mediated mitochondrial, and lysosomal pathway in Panc-28 cells, which resulted reversible by using the ROS scavenger vitamin C [47]. The expression of apoptosis-correlated proteins was also affected in cells exposed tooleanolic acid, including activation of caspases-3/9 and cleavage of PARP [50].

Further study revealed others mechanism of action of oleanolic acid, such as mitochondrial depolarization, release of cytochrome C, lysosomal membrane permeabilization and leakage of cathepinB [47].

MS-based lipidomic analysis of sphingolipids from metastatic pancreatic tissues revealed discrete and significant changes in specific ceramide molecule as compared to either pancreatic cancers without metastases or non-cancerous pancreatitis specimens [51]. Sphingosine and sphingosine-1-phosphate (S1P) manifest diverse biological, biochemical and biophysical functions that mediate, in part, cellular proliferation, migration, tumorigenesis, apoptosis and inflammation [52, 53] and are implicated in tumor growth of pancreatic stellate cells [54]. Mechanistically, sphingosine and S1P are typically considered pro-inflammatory, pro-mitogenic, and/or chemotaxic lipids that may facilitate the progression of pancreatic cancer, and it has been recognized and accepted by most researchers that sphingosine kinase acts like an oncogene and is upregulated in many human cancers [55]. Out of three sphingolipids included in our study one was sphingosine which resulted significantly higher (0.24±0.04 μM, mean±SD) in PC patients than normal controls (0.09±0.08 μM, mean±SD). Our data were keeping with a previous study performed by Jiang et al., who reported elevated levels in plasma of PC subjects of ceramide metabolites, including phosphorylated (sphingosine- and sphinganine-1-phosphate) and glycosylated (cerebroside) species [51]. The deregulation of sphingolipid metabolism was associated with more advanced and aggressive behavior of the cancer. The estimation of the Area Under the Curve (AUC) for sphingosine was 0.942 (0.877-1.000) (95%CI), showing a good sensitivity (92.5%) and specificity (97.5%) in the discrimination between healthy controls and pancreatic cancer patients.

It is well known that, to fulfill the increased energy requirements, pancreatic tumor cells secrete cytokines/factors inducing muscle and fat degradation in PC patients, a condition known as cancer cachexia. A high-fat and low-carbohydrate diet that elevates circulating levels of ketone bodies (i.e., acetoacetate, β-hydroxybutyrate, and acetone) diminish the overall energetic health of pancreatic cancer cells by reducing glucose uptake, lactate release, glutamine uptake, cellular ATP content, and ROS levels [56].

Epidemiological studies have demonstrated a positive correlation between ingestion of a western-style high fat diet and the incidence of pancreatic cancer [57].

Diets with a high fat content stimulate bile secretion, which increases the availability of cholesterol, bile acids and their metabolites.

Pancreatic cancer frequently causes obstructive jaundice and elevates bile acid levels in serum. Bile acids are steroid metabolites of cholesterol functioning as trophic factors for gut epithelium and as detergents for absorption of cholesterol and fat-soluble vitamins. Cytotoxicity of bile acid stems from its hydrophobic features and hydrophobic-hydrophilic balance in serum. Lipophilic unconjugated bile acids have been shown to flip-flap rapidly across artificial lipid bilayers, so that unconjugated bile acids might enter the cytosol of cells even at a low concentration. It has also been demonstrated that chenodeoxycholic acid (CDCA) administration in rat colon could enhance cell membrane phospholipid turnover [58], showing a significant increase of glycine and taurine conjugates species in patients with extrahepatic cholestasis. Finally, hydrophobic bile acids, such as DCA, could damage cells by lysing membranes and impairing mitochondrial function as well as increasing the generation of reactive oxygen radicals which can cause membrane lipid peroxidation and attack nucleic acids. The metabolic analysis in our investigation indicated that free bile acids in plasma of PC patients were significantly lower than controls. Instead, the concentrations of conjugated (glycine and taurine) cholic and chenodeoxycholic acids were significantly higher in PC group than controls, resembling the elevation of some bile acids in jaundiced serum of PC subjects that seems to inhibit pancreatic cell growth and proliferation [59].

In summary our mass spectrometry-based metabolomic profiling of pancreatic cancer patients and normal subjects allowed us to identify four metabolites (oleanoic acid, palmitic acid, taurochenodeoxycholate and d-sphingosine) with highly discriminative potential. Palmitic acid has a better discriminating ability in comparison to the pretherapeutic tumor marker CA 19-9 suggesting that, the joint evaluation of both pretherapeutic tumor markers CA19-9 and palmitic acid levels should be recommended in order to significantly reduce the probability to detect false positives and it could be used to improve the prognostic prediction in patients with pancreatic cancer.

## MATERIALS AND METHODS

### Patients and outline of the study

Forty patients with a confirmed diagnosis of PDAC and forty healthy blood donors (controls), referred to the “Casa Sollievo della Sofferenza” Hospital of San Giovanni Rotondo, Italy, were enrolled in the study. All patients signed an informed consent approved by the local Ethics Committee. The consent included collection of serum sample and clinical-pathological patient's features. The latter are reported in the Table A of the twin paper [69]. 50μl of serum sample was available for each subjects, two different but complementary targeted metabolomics approaches (commercial and custom-made quantitative methods) were used in order to analyze a large number of polar and hydrophobic metabolites in serum samples of healthy and pancreatic cancer' patients. The first one was a combination of direct injection and LC-MS/MS assay (Absolute IDQ p180 kit, Biocrates Life Sciences AG, Innsbruck, Austria) for the analysis of amino acids and biogenic amines, acylcarnitines, glycerophospholipids and hexoses. The second one was a “custom made” LC-MS/MS assay for the analysis of bile acids, fatty acids, sterols, glycerolipids, glycerophospholipids and sphingolipids. All details about preparation of standard solutions, validation of the method and instrumental parameters are described in the Supplementary Electronic Material (Supplementary Table 4–7).

### Chemicals and reagents

The AbsoluteIDQ^®^ p180 Kit was purchased from Biocrates Life Sciences AG, Innsbruck, Austria. All chemical standards of lipids were purchased from Aldrich-Fluka-Sigma S.r.l. (Milan, Italy). Labeled internal standards: D3-octanoyl L-carnitineHCl, D3-hexadecanoyl L-carnitineHCl, D3-acetyl L-carnitineHCl, D3-L-carnitine HCl, D3-eicosanoic acid, D3-octadecanoic acid (D3-stearic acid) and D3-tetradecanoic acid (D3-myristic acid) were from CDN Isotope (Quebec, Canada); D7-cholesterol, D4-cholic acid, D4-chenodeoxycholic acid, D4-glycoursodeoxycholic acid, D4-glycocholic acid, D5-taurocholic acid, D5-taurodeoxycholic acid, D4-lithocholic acid were from Sigma–Aldrich (Sigma–Aldrich, Milan, Italy). All chemicals had a purity grade greater than 90% and were used without further purification. Acetonitrile (ACN), 2-propanol (IPA), methanol (CH3OH), chloroform (CHCl3) and triethylamine (TEA) were purchased from Sigma–Aldrich (Sigma–Aldrich, Milan, Italy). All these solvents were of LC-MS grade, water was MilliQ (Millipore, Vimodrone, Milan, Italy). Formic acid (HCOOH) and ammonium formate (NH4COOH) additive for LC–MS were from FLUKA (Sigma–Aldrich, Milan, Italy).

### Metabolomic measurements by Biocrates kit

The serum samples, from patients and controls, were collected after an overnight fast and stored in −80°C till analysis. A targeted metabolomic assay was performed in samples of serum from pancreatic cancer patients and healthy control subjects (n=80) using the BiocratesAbsoluteIDQ^®^ p180 Kit (BIOCRATES life Sciences AG, Innsbruck, Austria), as previously described [60, 61]. Briefly, the flow injection analysis (FIA) tandem mass spectrometry (MS/MS) method was used to quantify 180 known small molecule metabolites simultaneously by multiple reaction monitoring (MRM) using an Acquity UPLC chromatographic system (Waters, Millford, MA, USA) coupled with a Xevo TQ-MS mass spectrometer (Waters, Millford, MA, USA). Quantification of the metabolites was achieved by reference to appropriate internal standards. Reproducibility of the assay was performed on QC serum samples. Concentrations of all analysed metabolites were reported in μM. The metabolomics dataset contains 40 acylcarnitines (Cx:y), hydroxylacylcarnitines(C(OH)x:y) and dicarboxylacylcarnitines (Cx:y-DC); 40 amino acids and biogenic amines; one sugar; 10 sphingomyelins (SMx:y) and sphingomyelin-derivatives (SM(OH)x:y); and 90 glycerophospholipids (PC). Glycerophospholipids are differentiated with respect to the presence of ester (a) and ether (e) bonds in the glycerol moiety, where two letters (aa = diacyl, ae = acyl-alkyl) denote that two glycerol positions are bound to a fatty acid residue, while a single letter (a = acyl) indicates the presence of a single fatty acid residue. Lipid side chain composition is abbreviated as Cx:y, where x denotes the number of carbons in the side chain and y the number of double bonds. The full list of metabolites is presented in the supplementary material (Supplementary Table 1).

### Targeted lipids analysis

An Ostro™ 96-well plate (Waters, Milford, MA, U.S.A.) was used to extract from human sera of a series of metabolites belonging to different classes of lipids: bile acids, fatty acids, sterols, glycerolipids, glycerophospholipids and sphingolipids.

The sera samples (50 μL) were loaded into the well plate and spiked with two internal standard solutions (50 μL of 50 μM of labeled bile acids and 10 μL of 500 μM of labeled lipids), mixed with 500 μL of acetonitrile with 0.1% formic acid (v/v) and transferred to a 96-well vacuum manifold where vacuum suction was applied for extraction. It took less than 3 min for the precipitated sample to completely pass through the packed bed. This procedure was repeated twice. The resulting eluent was collected in a 96-well collection plate and dried using N2 gas at 40°C, and finally reconstituted in 50 μL of MeOH:H2O 50:50 (v/v). The precipitated material into the well plate was washed twice with 800μL of a solution of MeOH:CHCl_3_:TEA (45:45:10, v/v/v) and transferred to a 96-well vacuum manifold where vacuum suction was applied for the elution of the phospholipids retained in the first step. The resulting eluent was collected in a 96-well collection plate and was dried using N2 gas at 40°C, and then reconstituted in 50 μL of IPA:ACN:H2O (65:30:5, v/v/v). Reconstituted samples were transferred to glass autosampler vials with glass inserts (200 μL) and injected into the chromatographic system for analysis. LC-MS/MS analyses were performed using an UHPLC Dionex3000 (Thermo Fisher Scientific, Germany) coupled with an API 5500 triple quadruple mass spectrometer (Applied Biosystems/MDS Sciex, Toronto, Canada) equipped with an electrospray ion source (ESI) operating in polarity switching mode. The injection volume was 5 μL. Supplementary Figure 1 shows a representative extracted ion chromatogram (XIC) of all lipids in a serum QC sample. The method validation assays were performed according to the currently accepted US Food and Drug Administration (FDA) bioanalytical method validation guide [62]. More details about the analytical methods can be found in electronic supplementary material.

### Principal component analysis

Metabolite levels were transformed into a set of values of linearly uncorrelated components. The transformation was defined in such a way that the first component had the largest possible variance and each succeeding component, in turn, had the highest variance possible under the constraint that it is orthogonal to (i.e., uncorrelated with) the preceding components. First three principal components were plotted in two 3D plots in Figure 1.

### Bioinformatics analysis

Metabolite levels from different platforms were analyzed separately to avoid the introduction of platform-specific biases, due to diverse dynamic ranges, machine-bound sensitivity and accuracy. Three methods were used (described below) to determine their differential over-representations between diseased and healthy patients. The best result came from the consensus of the three outcomes and was candidate for downstream pathway and feature selection analyses.

### Differential metabolomics fingerprint

#### Sparse PLS-DA

Sparse Partial Least Square Discriminant Analysis (sPLS-DA) is a sparse version of the PLS method [63], which tackles the classification problem by Linear Discriminant Analysis (LDA). It is effectively used to both reduce ill-conditioned problems due to multicollinearity of high throughput data and to select relevant features that maximally discriminate between classes. It takes two block data matrices: Y of size (n×q), which is coded with dummy variables to indicate the class membership of the n samples, and X (n×p), which holds the features to be discriminated. PLS is used to simultaneously decompose X and Y into latent variables and associated loading vectors [64]. We recall here that all the latent variables methods (e.g. PLS and Principal Component Regression) assume that the studied systems are driven by a small number of n-dimensional vectors called latent variables and that these might be responsible, or at least be involved in, the underlying biological phenomenon [63]. Latent variables are linear combinations of the variables. Sparse PLS [63], advances from PLS because it incorporates variable selection into PLS by penalizing loading vectors simultaneously. PLS is principally designed for regression problems, it performs well for classification problems also. Combined with a LDA method and after selection of the wished number of discriminant vectors: H ≤ min (p, K−1), where p is the total number of variables and K is the number of classes, a p-dimensional data are projected onto a H-dimensional space spanned by the first H discriminant vectors, also known as dimensions in the case of sPLS, and the following function minimized:

$$\underset{a_{h}{,\;b}_{h}}{\min}∥M_{h}-a_{h}b_{h}^{\prime }{∥}^{2}+P_{\lambda }\left(a_{h}\right),$$

where ‖·‖^2^ is the Frobenius norm between the current cross product matrix (M_h_ = X^T^_h_Y_h_) and the loading vectors (a_h_, b_h_); P_λ_(a_h_) = sign (a_h_) (a_h_ − λ)_+_ is the soft-thresholding function that approximates the Lasso penalty function [65]. In other words, this minimization can be viewed as the minimization problem of the regression model (‖·‖^2^ is the sum of squares) penalized by P_λ_(·) which allows a variable selection by approximating the coefficients to zero values. The coefficients with values different from zero are considered into the model. In particular, in an sPLS-DA framework, the selected variables are those that best discriminate between two groups. The sPLS-DA procedure is implemented in the R package mixOmics (http://www.math.univ-toulouse.fr/∼biostat/mixOmics). The required X matrix was set with metabolite levels, while the qualitative response matrix Y was made of indicator variables for class membership (0 = diseased, 1 = healthy). The number of discriminant vectors (or components) H was set to 3, in order to plot results in a 3D space (Figure 1).

#### Greedy stepwise

This method (also known as the hill-climbing attribute selection approach) searches for an optimal subset of discriminant metabolites, greedily. It progresses forward or backward from the empty and the full sets, in order to find the metabolites that maximize a chosen evaluation metrics. It does not backtrack but stops when the addition/deletion of any remaining metabolite results in a decrease in evaluation. The considered metrics is the distance between the tumor and control classes (Figure 3).

#### Genetic search

The GeneticSearch algorithm manipulates information proper to genetics like population size, number of generations and probabilities of crossover and mutations, and works in six steps: initialization, evaluation, selection, recombination, mutation and replacement. In the initialization phase, GeneticSearch starts with a random sample of the candidate feature subsets. This sample set is known as population, and each candidate feature subset is known as chromosome in the GeneticSearch terminology. A chromosome is represented as a binary string of zeros and ones, where zero represents the absence of a feature in the chromosome, whereas one represents the presence of a feature. The size of a chromosome string corresponds to the number of features. In the evaluation phase, each chromosome is evaluated according to fitness function. In the selection phase, the probability of selecting a chromosome is computed by the following equation:

$$p_{i}=\frac{f_{i}}{\sum_{i=1}^{n}f_{i}}$$

where *i* is a chromosome, *f_i_* is the value of the fitness function for *i*, and *n* is the number of chromosomes, or size of the population. Now, the cumulative probability of each chromosome $q_{i}=\sum_{j=1}^{i}p_{j}$ is computed. A random number *r* is generated between 0 and 1. The first chromosome with *q_i_ > r* is selected, and forms part of the next generation of the population. The selection process is repeated *n* times. It is possible that a particular chromosome is selected more than once; so the selection phase creates copies of chromosomes with high fitness values relative to other chromosomes in the population. In the recombination phase, two randomly selected chromosomes are combined with a cross over probability *p_c_* to form offspring chromosomes. There are several recombination rules in the literature. The most common one is based on exchange of bits between chromosomes. In the mutation phase, individual bits of a chromosome are flipped with a mutation probability *p_m_*. In the final phase replacement, the initial population is replaced by the new generation, which was obtained by selection, recombination and mutation. We have applied GeneticSearch implemented in Weka (20) for finding the best number of metabolites that maximize the separation between diseased and healthy patients. We set the following parameters: Population size: 20; Number of generations: 20; Probability of crossover: 0.6; Probability of mutation: 0.033; Report frequency: 20; Random number seed: 1.

### Statistical analysis

Medians and lower-upper quartiles of metabolite levels were reported in patients with pancreatic cancer and in healthy donors, separately. To validate bioinformatics findings (i.e. the three methods for the identification of those metabolites which best discriminate pancreatic cancer patients from healthy donors), we used the random forest analysis (RF) [66] (see Random Forest analysis section). The discriminatory ability of each metabolite was assessed by estimating the Area Under the Receiver Operating Characteristic (ROC) curves (AUC), along with their 95%confidence intervals (95%CI), following DeLong method [67]. The optimal cut-off was assessed by jointly maximizing sensitivity and specificity. Sensitivity and specificity, computed at the optimal cut-off, were further reported. A p value <0.05 was considered for statistical significance. All statistical analyses were performed using SAS Release 9.3 (SAS Institute, Cary, NC, USA). For the RF analysis, R software (ver. 2.15, package: “randomforestSRC”) was used.

### Random forest analysis

This appealing method is widely used to perform: classification, regression and survival analyses identifying, among all available covariates (i.e., all metabolites), the ones which were most associated to a specific dependent variable of interest (i.e., subject's status: pancreatic cancer or healthy donor). This method is also known to provide extremely robust results, especially in context of high dimensional dataset. To our purpose, we built 100.000 trees. The training set used to grow each tree is a 0.632+ bootstrap resample of the observations [68]. Trees were allowed to grow to their full size without pruning. The best split at each node of the tree included into the forest was selected from a random subset of covariates. The left-out (i.e. “out of bag”) observations were then used to obtain the classification error of the considered tree. The RF goodness of fit was assessed averaging the individual tree classification errors. One of the most relevant strength of RF analysis is its capability to impute missing data. Prior to splitting a node, missing data for a variable is imputed by randomly drawing values from non-missing in-bag data. The purpose of this imputed data is to make it possible to assign cases to daughter r nodes in the event the node is split on a variable with missing data. Imputed data is however not used to calculate the split-statistic which uses non-missing data only. Following a node split, imputed data are reset to missing and the process is repeated until terminal nodes are reached. Missing data in terminal nodes are imputed using OOB non-missing terminal node data. Furthermore, the RF framework estimates the importance of each covariate achieved to discriminate the dependent variable by looking at how much the classification error increases when out of bag data for that variable are permuted, while all others are left unchanged. The variables' importance was ranked by assigning to each covariate a score based on the ability to predict correctly the dependent variable according to the increase of classification error when values of that covariate in a node were randomly permuted. The importance metric used was the Mean Decrease in Accuracy (MDA). The MDA is constructed by permuting the values of each variable of the test set, recording the prediction and comparing it with the un-permuted test set prediction of the variable. Therefore, it is the increase in the percentage of times a test set is misclassified when the variable is permuted.

## SUPPLEMENTARY FIGURES AND TABLES
















