# Supplementary material for: Metabolomic profile in pancreatic cancer patients: a consensus-based approach to identify highly discriminating metabolites
Source: Oncotarget. 2016 Jan 1;7(5):5815–29. doi: 10.18632/oncotarget.6808 (PMC4868723; doi:10.18632/oncotarget.6808)
Supplement: Supplementary file 1 [file oncotarget-07-5815-s001.pdf]

SUPPLEMENTARY FIGURE AND TABLES

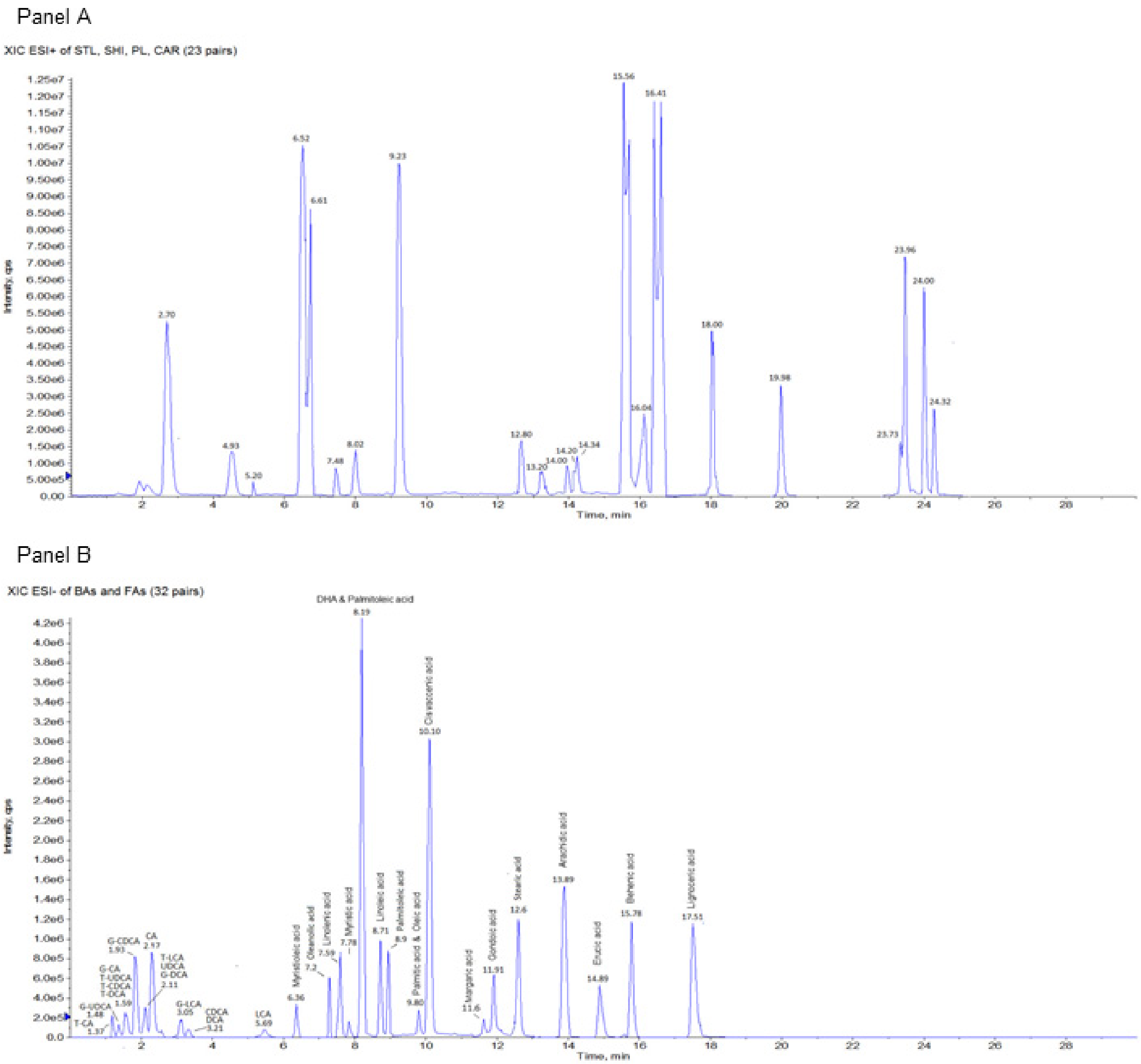

Supplementary Figure S1: Extracted ion chromatogram (XIC) of ESI positive and negative mode from a QC serum sample, expressed as percent relative abundance versus time in minutes. MRM functions are reported for each metabolite and its relative internal standard.

Supplementary Table S1: Medians (along with lower-upper quartiles) of metabolite levels (µmol/L) in patients with pancreatic cancer and in healthy donor

See supplementary Table 1

Supplementary Table S2: Variable importance (VIMP) and relative VIMP ranking

See supplementary Table 2

Supplementary Table S3: Estimation of the Area Under the Curve (AUC) for each metabolite, along with 95% confidence interval (95%CI), and the cut-off which best jointly maximize sensitivity (SE) and specificity (SP).

See supplementary Table 3

Supplementary Table S4: List of the analytical standards used for the LC-MS/MS method setting, with relative molecular formula and Chemspider ID.

See supplementary S4

Supplementary Table S5: Linearity parameters (range, equation, R2) obtained by using lipids standards in water and serum extracts along with evaluation of the matrix effects measured as 100% x (1 – slope water / slope serum)

See supplementary S5

Supplementary Table S6: Compound-specific instrumental parameters of the LC-ESI-MS/MS method for the analysis of lipids in serum samples. IS: isotope labeled internal standard, used for quantification

See supplementary S6

Supplementary Table S7: Method validation results for each quantified lipid by LC-ESI-MS/MS analysis

See supplementary S7
