# Supplementary material for: Metabolomic profile in pancreatic cancer patients: a consensus-based approach to identify highly discriminating metabolites
Source: Oncotarget. 2016 Jan 1;7(5):5815–29. doi: 10.18632/oncotarget.6808 (PMC4868723; doi:10.18632/oncotarget.6808)
Supplement: Supplementary file 2 [file oncotarget-07-5815-s002.docx]

**Supplementary Table 1.** Medians (along with lower-upper quartiles) of metabolite levels ( µmol/L) in patients with pancreatic cancer and in healthy donor

| **Metabolites** | **N of subjects** | **Healthy donors**  **(N=40)** | **Pancreatic cancer patients (N=40)** |
| --- | --- | --- | --- |
| C0 | 80 | 38.12 (33.81-45.51) | 41.45 (33.61-47.79) |
| C10:2 | 72 | 0.04 (0.03-0.06) | 0.06 (0.04-0.09) |
| C12-DC | 73 | 0.04 (0.03-0.04) | 0.04 (0.03-0.08) |
| C14:1 | 80 | 0.53 (0.41-0.63) | 0.29 (0.22-0.37) |
| C14:2 | 68 | 0.06 (0.04-0.08) | 0.05 (0.03-0.07) |
| C14:2-OH | 72 | 0.02 (0.01-0.03) | 0.02 (0.01-0.02) |
| C16 | 73 | 0.14 (0.10-0.18) | 0.11 (0.08-0.17) |
| C16-OH | 77 | 0.01 (0.01-0.02) | 0.02 (0.01-0.02) |
| C16:1 | 68 | 0.04 (0.03-0.05) | 0.05 (0.04-0.07) |
| C16:2 | 77 | 0.02 (0.01-0.02) | 0.02 (0.01-0.03) |
| C16:2-OH | 74 | 0.02 (0.01-0.03) | 0.02 (0.02-0.03) |
| C18 | 70 | 0.06 (0.05-0.09) | 0.06 (0.04-0.08) |
| C18:1 | 79 | 0.23 (0.18-0.32) | 0.24 (0.18-0.30) |
| C18:1-OH | 75 | 0.02 (0.01-0.03) | 0.03 (0.02-0.04) |
| C18:2 | 69 | 0.09 (0.07-0.11) | 0.07 (0.05-0.09) |
| C2 | 80 | 4.78 (3.75-5.56) | 6.09 (4.11-7.88) |
| C3 | 40 | 0.26 (0.18-0.31) | 0.28 (0.20-0.38) |
| C3-DC (C4-OH) | 77 | 0.08 (0.06-0.10) | 0.11 (0.09-0.15) |
| C3-OH | 72 | 0.04 (0.03-0.05) | 0.03 (0.03-0.05) |
| C3:1 | 77 | 0.02 (0.01-0.02) | 0.02 (0.01-0.02) |
| C4:1 | 74 | 0.03 (0.02-0.03) | 0.03 (0.02-0.04) |
| C5 | 73 | 0.12 (0.09-0.16) | 0.13 (0.11-0.19) |
| C5-OH (C3-DC-M) | 76 | 0.07 (0.06-0.11) | 0.11 (0.08-0.12) |
| C5:1 | 72 | 0.03 (0.02-0.04) | 0.02 (0.02-0.03) |
| C5:1-DC | 72 | 0.03 (0.02-0.04) | 0.03 (0.02-0.03) |
| C6:1 | 68 | 0.06 (0.04-0.08) | 0.05 (0.04-0.06) |
| C7-DC | 73 | 0.04 (0.03-0.04) | 0.03 (0.02-0.04) |
| C9 | 76 | 0.07 (0.05-0.08) | 0.05 (0.03-0.07) |
| Ala | 80 | 506.25 (433.15-564.80) | 555.20 (411.30-795.25) |
| Arg | 80 | 166.85 (139.05-189.85) | 122.90 (103.80-152.85) |
| Asn | 74 | 59.25 (56.00-61.50) | 61.95 (54.75-67.40) |
| Gln | 80 | 295.10 (265.20-400.25) | 281.25 (208.60-396.30) |
| Glu | 80 | 111.65 (88.00-150.05) | 110.35 (80.25-177.45) |
| Gly | 80 | 372.60 (340.30-412.25) | 263.80 (235.35-300.20) |
| His | 80 | 128.95 (115.20-141.90) | 77.85 (62.70-93.70) |
| Ile | 80 | 100.85 (83.85-112.85) | 89.75 (71.45-108.00) |
| Leu | 57 | 56.80 (37.10-81.45) | 65.90 (26.90-101.00) |
| Lys | 80 | 251.75 (218.45-287.60) | 201.80 (165.75-252.65) |
| Met | 80 | 35.15 (27.85-41.55) | 33.30 (27.45-41.25) |
| Orn | 80 | 130.30 (104.95-147.55) | 88.00 (78.75-106.65) |
| Phe | 80 | 137.25 (126.40-155.60) | 99.30 (83.05-120.60) |
| Pro | 80 | 239.10 (194.90-292.00) | 205.75 (164.35-273.20) |
| Ser | 78 | 166.50 (140.10-209.80) | 134.40 (92.00-181.20) |
| Thr | 80 | 173.90 (143.85-200.50) | 250.70 (160.50-299.90) |
| Trp | 80 | 75.85 (70.30-87.75) | 56.10 (37.55-63.55) |
| Tyr | 80 | 87.35 (72.90-103.05) | 68.65 (62.65-86.50) |
| Val | 80 | 278.20 (237.80-310.75) | 233.55 (189.65-294.25) |
| alpha-AAA | 41 | 9.50 (9.10-9.70) | 9.00 (9.00-9.40) |
| Creatinine | 80 | 115.00 (93.25-133.70) | 162.50 (123.35-219.55) |
| Kynurenine | 80 | 2.10 (1.20-2.70) | 1.90 (0.90-2.85) |
| Putrescine | 44 | 0.20 (0.20-0.25) | 0.40 (0.30-0.55) |
| SDMA | 59 | 0.50 (0.40-0.60) | 0.70 (0.40-1.00) |
| Serotonin | 74 | 0.40 (0.20-0.50) | 0.70 (0.30-1.20) |
| Spermidine | 73 | 0.30 (0.30-0.40) | 0.40 (0.30-0.50) |
| t4-OH-Pro | 80 | 9.40 (7.35-12.45) | 10.80 (7.55-16.55) |
| Taurine | 80 | 199.15 (170.60-220.25) | 170.80 (120.45-209.75) |
| lysoPC a C16:0 | 80 | 247.69 (230.57-301.71) | 90.58 (55.93-120.12) |
| lysoPC a C16:1 | 80 | 5.92 (4.97-6.62) | 2.65 (2.15-3.56) |
| lysoPC a C17:0 | 80 | 4.62 (3.88-5.78) | 1.56 (1.12-2.17) |
| lysoPC a C18:0 | 80 | 76.89 (67.11-93.87) | 19.64 (12.83-29.16) |
| lysoPC a C18:1 | 80 | 45.38 (40.46-54.78) | 21.39 (15.09-25.56) |
| lysoPC a C18:2 | 80 | 39.22 (32.95-46.31) | 15.85 (11.63-23.03) |
| lysoPC a C20:3 | 74 | 4.70 (3.94-6.12) | 2.40 (1.98-3.26) |
| lysoPC a C20:4 | 80 | 11.88 (10.26-15.07) | 5.66 (4.66-8.89) |
| lysoPC a C24:0 | 80 | 0.99 (0.83-1.34) | 0.76 (0.53-0.99) |
| lysoPC a C26:0 | 80 | 1.96 (1.56-2.39) | 1.33 (1.13-1.65) |
| lysoPC a C26:1 | 80 | 1.42 (1.14-1.73) | 0.97 (0.76-1.22) |
| lysoPC a C28:0 | 80 | 1.98 (1.64-2.31) | 1.47 (1.19-1.74) |
| lysoPC a C28:1 | 80 | 2.60 (1.93-3.19) | 1.67 (1.29-2.13) |
| PC aa C24:0 | 80 | 0.36 (0.29-0.44) | 0.27 (0.22-0.34) |
| PC aa C26:0 | 80 | 1.70 (1.40-2.11) | 1.56 (1.34-2.16) |
| PC aa C28:1 | 80 | 1.68 (1.43-2.05) | 1.29 (1.01-1.54) |
| PC aa C30:0 | 80 | 2.91 (2.47-3.46) | 2.78 (1.80-4.82) |
| PC aa C32:0 | 80 | 8.36 (7.36-9.58) | 9.43 (7.93-17.54) |
| PC aa C32:1 | 80 | 7.64 (5.28-9.57) | 10.31 (7.58-24.88) |
| PC aa C32:3 | 80 | 0.25 (0.21-0.30) | 0.18 (0.14-0.24) |
| PC aa C34:1 | 80 | 171.23 (136.78-191.80) | 182.68 (152.77-352.28) |
| PC aa C34:2 | 80 | 218.56 (190.84-255.56) | 208.50 (169.80-301.02) |
| PC aa C34:3 | 80 | 6.25 (4.74-7.35) | 5.90 (4.46-10.81) |
| PC aa C34:4 | 80 | 0.91 (0.74-1.13) | 0.55 (0.37-0.82) |
| PC aa C36:0 | 80 | 5.97 (5.22-6.72) | 4.17 (3.01-4.75) |
| PC aa C36:1 | 80 | 36.60 (29.52-42.02) | 29.58 (22.63-41.75) |
| PC aa C36:2 | 80 | 140.91 (126.66-166.38) | 107.47 (83.00-151.12) |
| PC aa C36:3 | 80 | 88.69 (73.65-100.99) | 72.42 (52.17-95.19) |
| PC aa C36:4 | 80 | 119.06 (101.77-146.53) | 112.85 (94.78-164.93) |
| PC aa C36:5 | 80 | 8.03 (6.55-11.66) | 7.02 (4.77-8.90) |
| PC aa C36:6 | 80 | 0.54 (0.41-0.62) | 0.32 (0.29-0.58) |
| PC aa C38:0 | 80 | 2.29 (2.07-2.86) | 1.82 (1.28-2.20) |
| PC aa C38:3 | 80 | 33.99 (29.24-41.47) | 24.05 (16.71-36.45) |
| PC aa C38:4 | 80 | 64.81 (54.49-82.84) | 53.47 (44.22-69.84) |
| PC aa C38:5 | 80 | 28.08 (22.73-32.84) | 22.98 (17.98-26.73) |
| PC aa C38:6 | 80 | 48.62 (38.63-58.43) | 39.97 (27.46-57.94) |
| PC aa C40:1 | 59 | 0.53 (0.48-0.62) | 0.48 (0.46-0.55) |
| PC aa C40:2 | 80 | 0.55 (0.46-0.67) | 0.46 (0.35-0.61) |
| PC aa C40:3 | 80 | 0.53 (0.48-0.64) | 0.49 (0.36-0.64) |
| PC aa C40:4 | 80 | 2.46 (2.10-3.12) | 1.92 (1.54-2.60) |
| PC aa C40:5 | 80 | 6.17 (4.87-7.44) | 4.71 (3.81-6.00) |
| PC aa C40:6 | 80 | 15.25 (11.84-19.27) | 12.27 (8.99-15.61) |
| PC aa C42:0 | 80 | 0.44 (0.35-0.55) | 0.38 (0.29-0.44) |
| PC aa C42:1 | 80 | 0.28 (0.23-0.33) | 0.19 (0.16-0.23) |
| PC aa C42:2 | 80 | 0.25 (0.21-0.28) | 0.17 (0.14-0.24) |
| PC aa C42:4 | 80 | 0.23 (0.19-0.27) | 0.20 (0.16-0.25) |
| PC aa C42:5 | 80 | 0.28 (0.23-0.35) | 0.22 (0.18-0.28) |
| PC aa C42:6 | 80 | 0.38 (0.33-0.44) | 0.29 (0.24-0.38) |
| PC ae C30:0 | 78 | 0.53 (0.45-0.66) | 0.41 (0.36-0.57) |
| PC ae C30:1 | 80 | 0.47 (0.41-0.54) | 0.39 (0.31-0.45) |
| PC ae C30:2 | 79 | 0.32 (0.26-0.39) | 0.23 (0.19-0.27) |
| PC ae C32:1 | 80 | 1.82 (1.60-2.14) | 1.54 (1.25-2.09) |
| PC ae C32:2 | 80 | 0.47 (0.42-0.58) | 0.38 (0.30-0.44) |
| PC ae C34:0 | 80 | 1.04 (0.92-1.22) | 1.01 (0.74-1.59) |
| PC ae C34:1 | 80 | 6.05 (5.22-7.25) | 6.33 (4.74-10.94) |
| PC ae C34:2 | 80 | 6.59 (5.34-7.62) | 4.57 (3.26-6.18) |
| PC ae C34:3 | 80 | 4.19 (3.40-4.64) | 2.29 (1.71-3.12) |
| PC ae C36:0 | 80 | 1.55 (1.28-1.82) | 1.23 (0.95-1.95) |
| PC ae C36:1 | 80 | 9.30 (7.91-10.40) | 7.86 (6.15-11.53) |
| PC ae C36:2 | 80 | 7.79 (6.38-8.64) | 6.30 (5.18-8.89) |
| PC ae C36:3 | 80 | 4.71 (4.07-5.46) | 3.04 (2.29-3.94) |
| PC ae C36:4 | 80 | 10.77 (9.51-12.49) | 7.31 (5.79-9.93) |
| PC ae C36:5 | 80 | 7.41 (6.57-9.02) | 5.71 (4.44-7.08) |
| PC ae C38:0 | 80 | 1.31 (1.11-1.66) | 1.01 (0.69-1.57) |
| PC ae C38:1 | 80 | 4.47 (4.01-5.28) | 2.46 (1.97-3.41) |
| PC ae C38:2 | 80 | 3.42 (2.99-4.02) | 2.24 (1.71-3.01) |
| PC ae C38:3 | 80 | 4.87 (4.22-5.53) | 3.42 (2.73-4.81) |
| PC ae C38:4 | 80 | 9.25 (7.72-10.72) | 6.72 (5.76-9.15) |
| PC ae C38:5 | 80 | 12.08 (9.76-14.02) | 9.43 (7.79-11.42) |
| PC ae C38:6 | 80 | 4.43 (3.98-5.30) | 3.14 (2.61-3.91) |
| PC ae C40:1 | 80 | 1.33 (1.16-1.50) | 0.86 (0.66-1.10) |
| PC ae C40:2 | 80 | 1.51 (1.30-1.76) | 1.22 (0.94-1.59) |
| PC ae C40:3 | 80 | 1.72 (1.52-2.03) | 1.13 (0.95-1.48) |
| PC ae C40:4 | 80 | 2.23 (1.92-2.52) | 1.53 (1.21-1.94) |
| PC ae C40:5 | 80 | 3.15 (2.79-3.89) | 2.44 (1.93-3.01) |
| PC ae C40:6 | 80 | 3.04 (2.75-3.66) | 2.43 (1.94-2.82) |
| PC ae C42:1 | 80 | 0.34 (0.28-0.41) | 0.28 (0.24-0.42) |
| PC ae C42:2 | 80 | 0.46 (0.41-0.55) | 0.35 (0.26-0.43) |
| PC ae C42:3 | 80 | 0.67 (0.55-0.74) | 0.40 (0.35-0.53) |
| PC ae C42:4 | 80 | 0.73 (0.62-0.83) | 0.52 (0.43-0.67) |
| PC ae C42:5 | 80 | 1.60 (1.47-2.01) | 1.32 (1.11-1.73) |
| PC ae C44:3 | 79 | 0.14 (0.12-0.17) | 0.11 (0.09-0.15) |
| PC ae C44:4 | 80 | 0.33 (0.28-0.40) | 0.25 (0.20-0.30) |
| PC ae C44:5 | 80 | 1.38 (1.23-1.64) | 1.17 (0.98-1.49) |
| PC ae C44:6 | 80 | 0.80 (0.71-0.96) | 0.65 (0.52-0.84) |
| SM (OH) C14:1 | 80 | 4.03 (3.44-4.91) | 3.70 (2.87-4.49) |
| SM (OH) C16:1 | 80 | 1.94 (1.66-2.39) | 2.01 (1.41-2.37) |
| SM (OH) C22:1 | 73 | 11.56 (8.57-14.07) | 3.34 (2.80-5.67) |
| SM (OH) C22:2 | 78 | 8.52 (6.56-10.98) | 5.30 (3.38-7.97) |
| SM C16:0 | 80 | 70.83 (61.72-78.53) | 65.13 (56.17-75.18) |
| SM C16:1 | 80 | 9.61 (8.69-11.47) | 8.74 (7.03-9.78) |
| SM C18:0 | 80 | 18.14 (14.70-21.03) | 19.06 (13.84-21.81) |
| SM C18:1 | 80 | 7.35 (5.98-8.75) | 7.04 (5.08-8.74) |
| SM C24:0 | 80 | 20.48 (19.39-23.01) | 13.05 (10.49-16.51) |
| SM C24:1 | 50 | 12.88 (6.88-23.59) | 31.90 (21.29-41.71) |
| H1 | 80 | 3458.43 (3073.03-3671.93) | 4969.39 (3977.38-6263.23) |
| Progesteron | 80 | 0.07 (0.06-0.09) | 0.17 (0.16-0.18) |
| 5-α-Cholestane | 80 | 0.79 (0.61-0.95) | 1.73 (1.16-2.92) |
| Chol_Epoxide | 80 | 0.56 (0.51-0.61) | 1.99 (1.88-2.12) |
| 1,2dilinoleoyl_PC | 80 | 34.21 (22.49-51.63) | 5.24 (3.27-9.96) |
| 1,2dioleoyl_GLP_Na2 | 80 | 1.96 (1.95-1.99) | 5.38 (5.36-5.39) |
| D-sphingosine | 80 | 0.24 (0.22-0.27) | 0.08 (0.05-0.12) |
| C16-CAR2 | 76 | 0.08 (0.08-0.09) | 0.05 (0.04-0.06) |
| Oleoyl-CAR | 76 | 1.65 (1.64-1.67) | 0.13 (0.11-0.15) |
| Lanosterol | 80 | 42.86 (38.28-56.89) | 132.71 (122.89-144.50) |
| CER_893_1 | 80 | 0.63 (0.48-0.74) | 0.87 (0.63-1.46) |
| 1-palmitoyl-sn-glycero-3PC | 80 | 282.17 (253.69-298.34) | 104.43 (64.62-142.71) |
| BSitosterol | 80 | 4.92 (4.49-5.70) | 3.83 (2.82-5.06) |
| glyceryltrioleate1 | 80 | 68.65 (50.86-92.08) | 75.75 (60.58-101.90) |
| 1,2dioleoyl_PE | 76 | 0.15 (0.10-0.45) | 1.01 (0.81-1.31) |
| cis-vaccenic_acid | 80 | 50.06 (35.49-65.89) | 75.24 (52.46-95.91) |
| ArachidicAcid | 80 | 2.16 (1.92-2.42) | 3.99 (3.73-4.36) |
| erucic acid | 80 | 0.68 (0.66-0.72) | 2.99 (2.84-3.09) |
| StearicAcid | 80 | 77.55 (74.16-80.84) | 154.08 (143.86-163.30) |
| PalmiticAcid | 80 | 321.50 (290.64-358.20) | 15.82 (15.28-16.93) |
| LinoleicAcid | 80 | 26.04 (22.80-41.25) | 38.97 (23.05-60.56) |
| DocosahexaenoicAcid | 80 | 3.14 (2.19-4.34) | 5.76 (4.84-7.73) |
| PalmitoleicAcid | 80 | 14.60 (9.64-19.76) | 31.47 (21.51-45.46) |
| BehenicAcid | 80 | 1.90 (1.86-2.57) | 0.77 (0.54-1.18) |
| MyristicAcid | 80 | 22.79 (19.74-28.45) | 25.65 (22.02-27.45) |
| LinolenicAcid | 80 | 4.62 (3.89-5.86) | 3.17 (2.46-4.46) |
| OleicAcid | 80 | 103.37 (82.20-129.53) | 126.16 (96.99-161.52) |
| MyristoleicAcid | 80 | 1.53 (1.11-2.05) | 1.29 (0.88-1.61) |
| LignocericAcid | 80 | 0.26 (0.19-0.52) | 4.14 (3.96-4.21) |
| MargaricAcid | 80 | 3.28 (2.78-4.21) | 6.63 (5.38-7.72) |
| oleanolic acid | 80 | 0.73 (0.64-0.79) | 8.61 (7.60-9.88) |
| tripentadecanoate TG15 | 80 | 2.77 (1.09-3.41) | 7.97 (6.79-9.79) |
| Glyceryltrilinoleate1 | 80 | 6.98 (4.40-8.91) | 26.74 (18.90-42.31) |
| glyceryltripalmitoleate1 | 80 | 0.67 (0.55-0.94) | 0.82 (0.57-1.24) |
| 1linoleoyl-rac-GL | 80 | 4.63 (4.58-4.72) | 2.61 (2.17-3.97) |
| 1oleoyl_rac_GL | 80 | 13.01 (12.93-13.13) | 9.51 (9.44-9.86) |
| 1monopalmitoleoyl-rac-GL1 | 80 | 10.34 (10.27-10.48) | 15.18 (15.15-15.25) |
| betaSitosterolglucoside | 40 | 1.88 (1.54-2.39) | N.A. |
| desmosterol1 | 80 | 0.44 (0.39-0.48) | 0.45 (0.40-0.60) |
| CHOLESTEROL2 | 76 | 259.97 (229.60-299.02) | 373.64 (301.61-490.58) |
| CA | 80 | 0.51 (0.47-0.81) | 0.15 (0.10-0.52) |
| CDCA | 80 | 0.48 (0.33-0.94) | 0.23 (0.16-0.77) |
| DCA | 80 | 0.33 (0.18-0.47) | 0.05 (0.02-0.34) |
| UDCA | 80 | 0.09 (0.05-0.16) | 0.04 (0.03-0.07) |
| LCA | 79 | 0.18 (0.17-0.19) | 0.17 (0.16-0.19) |
| G-CA | 80 | 0.29 (0.22-0.40) | 1.08 (0.24-5.31) |
| G-CDCA | 80 | 0.38 (0.21-0.56) | 1.85 (0.77-3.80) |
| G-DCA | 80 | 0.12 (0.06-0.27) | 0.26 (0.09-0.61) |
| G-UDCA | 80 | 0.13 (0.07-0.20) | 0.15 (0.10-0.24) |
| G-LCA | 79 | 0.03 (0.01-0.04) | 0.04 (0.03-0.07) |
| T-CA | 80 | 0.13 (0.11-0.14) | 0.26 (0.13-2.46) |
| T-CDCA | 80 | 0.06 (0.04-0.09) | 0.91 (0.37-5.24) |
| T-DCA | 80 | 0.15 (0.13-0.17) | 0.36 (0.13-1.17) |
| T-UDCA | 70 | 0.04 (0.04-0.05) | 0.03 (0.02-0.17) |
| T-LCA | 69 | 0.41 (0.41-0.42) | 0.03 (0.03-0.03) |
