# Supplementary material for: Metabolomic profile in pancreatic cancer patients: a consensus-based approach to identify highly discriminating metabolites
Source: Oncotarget. 2016 Jan 1;7(5):5815–29. doi: 10.18632/oncotarget.6808 (PMC4868723; doi:10.18632/oncotarget.6808)
Supplement: Supplementary file 3 [file oncotarget-07-5815-s003.docx]

**Supplementary Table 2**. Variable importance (VIMP) and relative VIMP ranking

| **Metabolite** | **VIMP** | **Relative VIMP** | |
| --- | --- | --- | --- |
| PalmiticAcid | 0.02980997 | | 100.0% |
| 1,2dioleoyl_GLP_Na2 | 0.02976101 | | 99.8% |
| Lanosterol | 0.02907528 | | 97.5% |
| LignocericAcid | 0.02896808 | | 97.2% |
| 1oleoyl_rac_GL | 0.02879624 | | 96.6% |
| Chol_Epoxide | 0.02866469 | | 96.2% |
| erucic acid | 0.02861594 | | 96.0% |
| T-LCA | 0.02849319 | | 95.6% |
| Oleoyl-CAR | 0.02840777 | | 95.3% |
| oleanolic acid | 0.02839765 | | 95.3% |
| BehenicAcid | 0.02047500 | | 68.7% |
| lysoPC a C18:0 | 0.01861972 | | 62.5% |
| Progesteron | 0.01826844 | | 61.3% |
| StearicAcid | 0.01691486 | | 56.7% |
| 1monopalmitoleoyl-rac-GL1 | 0.01587736 | | 53.3% |
| lysoPC a C16:0 | 0.01180564 | | 39.6% |
| T-CDCA | 0.01025462 | | 34.4% |
| tripentadecanoate TG15 | 0.00974178 | | 32.7% |
| lysoPC a C17:0 | 0.00718695 | | 24.1% |
| 1-palmitoyl-sn-glycero-3PC | 0.00431306 | | 14.5% |
| ArachidicAcid | 0.00423109 | | 14.2% |
| D-sphingosine | 0.00416883 | | 14.0% |
| Glyceryltrilinoleate1 | 0.00412332 | | 13.8% |
| lysoPC a C18:2 | 0.00200377 | | 6.7% |
| 1,2dioleoyl_PE | 0.00181137 | | 6.1% |
| SM (OH) C22:1 | 0.00154820 | | 5.2% |
| 1,2dilinoleoyl_PC | 0.00148952 | | 5.0% |
| MargaricAcid | 0.00145928 | | 4.9% |
| lysoPC a C18:1 | 0.00142713 | | 4.8% |
| G-CDCA | 0.00099002 | | 3.3% |
| 1linoleoyl-rac-GL | 0.00075887 | | 2.5% |
| His | 0.00072814 | | 2.4% |
| Putrescine | 0.00040187 | | 1.3% |
| G-LCA | 0.00038087 | | 1.3% |
| lysoPC a C16:1 | 0.00028382 | | 1.0% |
| DocosahexaenoicAcid | 0.00023951 | | 0.8% |
| C16-CAR2 | 0.00021574 | | 0.7% |
| lysoPC a C20:3 | 0.00021126 | | 0.7% |
| Trp | 0.00017000 | | 0.6% |
| SM C24:0 | 0.00014790 | | 0.5% |
| 5-α-Cholestane | 0.00012734 | | 0.4% |
| SM C24:1 | 0.00012096 | | 0.4% |
| CA | 0.00012016 | | 0.4% |
| lysoPC a C20:4 | 0.00011902 | | 0.4% |
| C14:1 | 0.00011351 | | 0.4% |
| PC ae C38:1 | 0.00011004 | | 0.4% |
| betaSitosterolglucoside | 0.00010867 | | 0.4% |
| Gly | 0.00010215 | | 0.3% |
| PC aa C36:0 | 0.00008757 | | 0.3% |
| PC ae C42:3 | 0.00007064 | | 0.2% |
| CHOLESTEROL2 | 0.00007044 | | 0.2% |
| PC ae C40:1 | 0.00005637 | | 0.2% |
| PalmitoleicAcid | 0.00004589 | | 0.2% |
| Phe | 0.00004566 | | 0.2% |
| alpha-AAA | 0.00004352 | | 0.1% |
| Thr | 0.00004100 | | 0.1% |
| Arg | 0.00003002 | | 0.1% |
| T-CA | 0.00002976 | | 0.1% |
| T-UDCA | 0.00002810 | | 0.1% |
| PC ae C38:2 | 0.00002705 | | 0.1% |
| PC ae C40:3 | 0.00002494 | | 0.1% |
| PC ae C36:3 | 0.00002044 | | 0.1% |
| Ala | 0.00001856 | | 0.1% |
| Creatinine | 0.00001852 | | 0.1% |
| PC ae C38:6 | 0.00001801 | | 0.1% |
| PC ae C34:3 | 0.00001740 | | 0.1% |
| PC aa C32:1 | 0.00001653 | | 0.1% |
| C5-OH (C3-DC-M) | 0.00001582 | | 0.1% |
| H1 | 0.00001354 | | 0.0% |
| UDCA | 0.00001326 | | 0.0% |
| G-CA | 0.00001323 | | 0.0% |
| PC ae C36:4 | 0.00001286 | | 0.0% |
| T-DCA | 0.00001235 | | 0.0% |
| PC aa C40:1 | 0.00001183 | | 0.0% |
| Lys | 0.00001034 | | 0.0% |
| SDMA | 0.00000973 | | 0.0% |
| SM (OH) C22:2 | 0.00000935 | | 0.0% |
| Taurine | 0.00000879 | | 0.0% |
| PC aa C34:3 | 0.00000878 | | 0.0% |
| PC aa C32:0 | 0.00000868 | | 0.0% |
| PC ae C40:6 | 0.00000860 | | 0.0% |
| CER_893_1 | 0.00000857 | | 0.0% |
| DCA | 0.00000830 | | 0.0% |
| Spermidine | 0.00000767 | | 0.0% |
| C16:2-OH | 0.00000765 | | 0.0% |
| Orn | 0.00000720 | | 0.0% |
| BSitosterol | 0.00000704 | | 0.0% |
| PC ae C42:5 | 0.00000701 | | 0.0% |
| PC ae C32:1 | 0.00000661 | | 0.0% |
| PC ae C44:4 | 0.00000660 | | 0.0% |
| PC ae C30:2 | 0.00000636 | | 0.0% |
| PC aa C38:0 | 0.00000630 | | 0.0% |
| lysoPC a C26:1 | 0.00000603 | | 0.0% |
| PC ae C44:6 | 0.00000584 | | 0.0% |
| PC aa C42:1 | 0.00000570 | | 0.0% |
| PC ae C34:1 | 0.00000569 | | 0.0% |
| PC aa C34:1 | 0.00000542 | | 0.0% |
| PC ae C38:0 | 0.00000508 | | 0.0% |
| C7-DC | 0.00000447 | | 0.0% |
| G-DCA | 0.00000401 | | 0.0% |
| PC ae C36:5 | 0.00000379 | | 0.0% |
| PC aa C42:0 | 0.00000362 | | 0.0% |
| PC ae C34:0 | 0.00000296 | | 0.0% |
| Val | 0.00000287 | | 0.0% |
| cis-vaccenic_acid | 0.00000286 | | 0.0% |
| Tyr | 0.00000286 | | 0.0% |
| PC ae C40:5 | 0.00000280 | | 0.0% |
| PC ae C40:4 | 0.00000279 | | 0.0% |
| C10:2 | 0.00000262 | | 0.0% |
| C9 | 0.00000257 | | 0.0% |
| PC ae C42:4 | 0.00000254 | | 0.0% |
| C18:1-OH | 0.00000230 | | 0.0% |
| C5:1 | 0.00000229 | | 0.0% |
| C16-OH | 0.00000224 | | 0.0% |
| Ser | 0.00000217 | | 0.0% |
| PC ae C36:1 | 0.00000207 | | 0.0% |
| PC aa C32:3 | 0.00000206 | | 0.0% |
| C16:2 | 0.00000198 | | 0.0% |
| LinoleicAcid | 0.00000198 | | 0.0% |
| PC aa C42:2 | 0.00000196 | | 0.0% |
| C6:1 | 0.00000191 | | 0.0% |
| PC aa C36:4 | 0.00000190 | | 0.0% |
| PC ae C38:3 | 0.00000151 | | 0.0% |
| PC ae C44:3 | 0.00000149 | | 0.0% |
| lysoPC a C28:1 | 0.00000149 | | 0.0% |
| MyristicAcid | 0.00000147 | | 0.0% |
| C0 | 0.00000135 | | 0.0% |
| Asn | 0.00000134 | | 0.0% |
| PC aa C42:4 | 0.00000133 | | 0.0% |
| MyristoleicAcid | 0.00000133 | | 0.0% |
| PC aa C36:5 | 0.00000131 | | 0.0% |
| PC ae C32:2 | 0.00000113 | | 0.0% |
| PC aa C42:5 | 0.00000112 | | 0.0% |
| PC aa C38:5 | 0.00000110 | | 0.0% |
| SM C18:0 | 0.00000082 | | 0.0% |
| PC ae C34:2 | 0.00000080 | | 0.0% |
| PC ae C44:5 | 0.00000074 | | 0.0% |
| C14:2-OH | 0.00000069 | | 0.0% |
| C18 | 0.00000065 | | 0.0% |
| lysoPC a C24:0 | 0.00000063 | | 0.0% |
| C12-DC | 0.00000061 | | 0.0% |
| PC aa C40:4 | 0.00000057 | | 0.0% |
| LCA | 0.00000056 | | 0.0% |
| PC aa C40:2 | 0.00000055 | | 0.0% |
| SM C18:1 | 0.00000053 | | 0.0% |
| C5:1-DC | 0.00000049 | | 0.0% |
| lysoPC a C28:0 | 0.00000037 | | 0.0% |
| PC aa C34:2 | 0.00000024 | | 0.0% |
| PC aa C42:6 | 0.00000013 | | 0.0% |
| PC aa C40:5 | 0.00000006 | | 0.0% |
| PC aa C36:6 | 0.00000000 | | 0.0% |
| PC ae C30:1 | 0.00000000 | | 0.0% |
| PC ae C40:2 | 0.00000000 | | 0.0% |
| PC ae C42:1 | 0.00000000 | | 0.0% |
| SM (OH) C14:1 | 0.00000000 | | 0.0% |
| C3-OH | 0.00000000 | | 0.0% |
| C3:1 | 0.00000000 | | 0.0% |
| C4:1 | 0.00000000 | | 0.0% |
| Gln | 0.00000000 | | 0.0% |
| Glu | 0.00000000 | | 0.0% |
| Leu | 0.00000000 | | 0.0% |
| Met | 0.00000000 | | 0.0% |
| Pro | 0.00000000 | | 0.0% |
| PC aa C24:0 | 0.00000000 | | 0.0% |
| PC aa C26:0 | 0.00000000 | | 0.0% |
| PC aa C36:1 | 0.00000000 | | 0.0% |
| C16:1 | 0.00000000 | | 0.0% |
| PC aa C38:4 | 0.00000000 | | 0.0% |
| PC aa C30:0 | -0.00000016 | | 0.0% |
| glyceryltrioleate1 | -0.00000031 | | 0.0% |
| C14:2 | -0.00000040 | | 0.0% |
| SM C16:1 | -0.00000043 | | 0.0% |
| t4-OH-Pro | -0.00000059 | | 0.0% |
| PC aa C40:6 | -0.00000066 | | 0.0% |
| PC aa C38:3 | -0.00000066 | | 0.0% |
| PC ae C36:0 | -0.00000067 | | 0.0% |
| C5 | -0.00000067 | | 0.0% |
| SM C16:0 | -0.00000068 | | 0.0% |
| PC ae C30:0 | -0.00000071 | | 0.0% |
| Kynurenine | -0.00000071 | | 0.0% |
| PC ae C38:4 | -0.00000084 | | 0.0% |
| C18:2 | -0.00000095 | | 0.0% |
| PC aa C38:6 | -0.00000110 | | 0.0% |
| C16 | -0.00000111 | | 0.0% |
| PC aa C36:2 | -0.00000128 | | 0.0% |
| desmosterol1 | -0.00000136 | | 0.0% |
| Ile | -0.00000138 | | 0.0% |
| glyceryltripalmitoleate1 | -0.00000148 | | 0.0% |
| PC aa C40:3 | -0.00000148 | | 0.0% |
| CDCA | -0.00000164 | | 0.0% |
| C3-DC (C4-OH) | -0.00000213 | | 0.0% |
| Serotonin | -0.00000220 | | 0.0% |
| PC ae C38:5 | -0.00000250 | | 0.0% |
| LinolenicAcid | -0.00000263 | | 0.0% |
| lysoPC a C26:0 | -0.00000293 | | 0.0% |
| C18:1 | -0.00000299 | | 0.0% |
| G-UDCA | -0.00000302 | | 0.0% |
| PC aa C36:3 | -0.00000333 | | 0.0% |
| PC aa C28:1 | -0.00000412 | | 0.0% |
| C2 | -0.00000492 | | 0.0% |
| SM (OH) C16:1 | -0.00000496 | | 0.0% |
| PC ae C42:2 | -0.00000504 | | 0.0% |
| OleicAcid | -0.00000506 | | 0.0% |
| PC aa C34:4 | -0.00000542 | | 0.0% |
| C3 | -0.00000584 | | 0.0% |
| PC ae C36:2 | -0.00000679 | | 0.0% |
