# Supplementary material for: Metabolomic profile in pancreatic cancer patients: a consensus-based approach to identify highly discriminating metabolites
Source: Oncotarget. 2016 Jan 1;7(5):5815–29. doi: 10.18632/oncotarget.6808 (PMC4868723; doi:10.18632/oncotarget.6808)
Supplement: Supplementary file 4 [file oncotarget-07-5815-s004.docx]

**Supplementary Table 3.** Estimation of the Area Under the Curve (AUC) for each metabolite, along with 95% confidence interval (95%CI), and the cut-off which best jointly maximize sensitivity (SE) and specificity (SP)

| **Metabolites** | **cut-off** | **SE** | **SP** | **AUC**  **(95%CI)** |
| --- | --- | --- | --- | --- |
| C0 | 43.448 | 0.475 | 0.675 | 0.542 (0.414-0.670) |
| C10:2 | 0.039 | 0.892 | 0.486 | 0.698 (0.577-0.819) |
| C12-DC | 0.055 | 0.436 | 0.853 | 0.618 (0.487-0.750) |
| C14:1 | 0.390 | 0.800 | 0.800 | 0.854 (0.767-0.942) |
| C14:2 | 0.034 | 0.297 | 0.903 | 0.569 (0.431-0.707) |
| C14:2-OH | 0.016 | 0.657 | 0.486 | 0.500 (0.363-0.636) |
| C16 | 0.134 | 0.611 | 0.595 | 0.595 (0.462-0.727) |
| C16-OH | 0.018 | 0.475 | 0.757 | 0.618 (0.491-0.745) |
| C16:1 | 0.044 | 0.639 | 0.625 | 0.652 (0.521-0.783) |
| C16:2 | 0.019 | 0.625 | 0.676 | 0.641 (0.516-0.766) |
| C16:2-OH | 0.019 | 0.789 | 0.500 | 0.662 (0.538-0.787) |
| C18 | 0.053 | 0.471 | 0.722 | 0.578 (0.442-0.715) |
| C18:1 | 0.214 | 0.625 | 0.462 | 0.492 (0.362-0.622) |
| C18:1-OH | 0.025 | 0.590 | 0.722 | 0.697 (0.579-0.815) |
| C18:2 | 0.082 | 0.676 | 0.594 | 0.639 (0.506-0.772) |
| C2 | 5.987 | 0.525 | 0.850 | 0.658 (0.535-0.780) |
| C3 | 0.315 | 0.409 | 0.778 | 0.551 (0.368-0.733) |
| C3-DC (C4-OH) | 0.099 | 0.667 | 0.763 | 0.730 (0.615-0.846) |
| C3-OH | 0.046 | 0.743 | 0.432 | 0.536 (0.400-0.672) |
| C3:1 | 0.015 | 0.737 | 0.487 | 0.584 (0.455-0.714) |
| C4:1 | 0.022 | 0.436 | 0.829 | 0.604 (0.474-0.734) |
| C5 | 0.174 | 0.368 | 0.857 | 0.587 (0.455-0.720) |
| C5-OH (C3-DC-M) | 0.078 | 0.838 | 0.564 | 0.681 (0.558-0.803) |
| C5:1 | 0.031 | 0.892 | 0.400 | 0.629 (0.497-0.760) |
| C5:1-DC | 0.038 | 0.838 | 0.371 | 0.551 (0.413-0.688) |
| C6:1 | 0.057 | 0.667 | 0.543 | 0.558 (0.417-0.700) |
| C7-DC | 0.032 | 0.632 | 0.714 | 0.665 (0.536-0.793) |
| C9 | 0.068 | 0.795 | 0.514 | 0.694 (0.575-0.812) |
| Ala | 660.750 | 0.425 | 0.925 | 0.576 (0.442-0.709) |
| Arg | 131.850 | 0.625 | 0.900 | 0.798 (0.699-0.896) |
| Asn | 61.750 | 0.528 | 0.816 | 0.560 (0.422-0.698) |
| Gln | 247.200 | 0.400 | 0.875 | 0.578 (0.449-0.707) |
| Glu | 79.450 | 0.250 | 0.875 | 0.489 (0.359-0.619) |
| Gly | 313.500 | 0.825 | 0.850 | 0.871 (0.790-0.952) |
| His | 104.900 | 0.850 | 0.950 | 0.949 (0.903-0.996) |
| Ile | 88.400 | 0.500 | 0.700 | 0.585 (0.459-0.712) |
| Leu | 58.250 | 0.600 | 0.563 | 0.488 (0.326-0.650) |
| Lys | 212.350 | 0.625 | 0.875 | 0.763 (0.656-0.869) |
| Met | 34.800 | 0.625 | 0.525 | 0.527 (0.397-0.658) |
| Orn | 92.350 | 0.575 | 0.925 | 0.838 (0.753-0.922) |
| Phe | 115.600 | 0.750 | 0.875 | 0.816 (0.715-0.918) |
| Pro | 222.200 | 0.600 | 0.650 | 0.618 (0.493-0.742) |
| Ser | 124.900 | 0.487 | 0.949 | 0.695 (0.575-0.814) |
| Thr | 244.250 | 0.525 | 0.975 | 0.672 (0.543-0.802) |
| Trp | 67.600 | 0.875 | 0.825 | 0.866 (0.780-0.951) |
| Tyr | 78.750 | 0.650 | 0.700 | 0.702 (0.587-0.816) |
| Val | 215.850 | 0.425 | 0.925 | 0.673 (0.551-0.794) |
| alpha-AAA | 9.050 | 0.545 | 0.789 | 0.718 (0.562-0.874) |
| Creatinine | 145.500 | 0.725 | 0.850 | 0.785 (0.678-0.892) |
| Kynurenine | 1.050 | 0.300 | 0.850 | 0.530 (0.401-0.659) |
| Putrescine | 0.250 | 0.821 | 0.750 | 0.857 (0.758-0.956) |
| SDMA | 0.650 | 0.515 | 0.923 | 0.696 (0.561-0.831) |
| Serotonin | 0.550 | 0.568 | 0.811 | 0.650 (0.517-0.783) |
| Spermidine | 0.450 | 0.382 | 0.949 | 0.690 (0.576-0.804) |
| t4-OH-Pro | 10.200 | 0.550 | 0.650 | 0.581 (0.453-0.708) |
| Taurine | 150.400 | 0.400 | 0.950 | 0.643 (0.517-0.770) |
| lysoPC a C16:0 | 156.900 | 0.950 | 1.000 | 0.997 (0.991-1.000) |
| lysoPC a C16:1 | 3.910 | 0.825 | 0.925 | 0.898 (0.817-0.978) |
| lysoPC a C17:0 | 2.718 | 0.950 | 0.975 | 0.990 (0.976-1.000) |
| lysoPC a C18:0 | 48.289 | 1.000 | 0.975 | 0.999 (0.996-1.000) |
| lysoPC a C18:1 | 33.377 | 0.875 | 0.975 | 0.964 (0.928-1.000) |
| lysoPC a C18:2 | 28.270 | 0.925 | 0.925 | 0.947 (0.893-1.000) |
| lysoPC a C20:3 | 3.438 | 0.824 | 0.900 | 0.885 (0.796-0.973) |
| lysoPC a C20:4 | 9.011 | 0.775 | 0.900 | 0.874 (0.794-0.954) |
| lysoPC a C24:0 | 0.821 | 0.600 | 0.775 | 0.713 (0.600-0.826) |
| lysoPC a C26:0 | 1.625 | 0.750 | 0.725 | 0.766 (0.658-0.875) |
| lysoPC a C26:1 | 1.051 | 0.650 | 0.850 | 0.766 (0.657-0.874) |
| lysoPC a C28:0 | 1.729 | 0.750 | 0.675 | 0.729 (0.616-0.843) |
| lysoPC a C28:1 | 1.868 | 0.675 | 0.775 | 0.762 (0.653-0.870) |
| PC aa C24:0 | 0.343 | 0.775 | 0.575 | 0.702 (0.585-0.819) |
| PC aa C26:0 | 1.732 | 0.650 | 0.500 | 0.534 (0.405-0.663) |
| PC aa C28:1 | 1.357 | 0.650 | 0.850 | 0.750 (0.638-0.862) |
| PC aa C30:0 | 2.462 | 0.475 | 0.775 | 0.507 (0.370-0.644) |
| PC aa C32:0 | 13.996 | 0.325 | 0.975 | 0.653 (0.530-0.776) |
| PC aa C32:1 | 8.398 | 0.675 | 0.700 | 0.726 (0.614-0.837) |
| PC aa C32:3 | 0.205 | 0.675 | 0.800 | 0.728 (0.610-0.845) |
| PC aa C34:1 | 258.054 | 0.325 | 0.950 | 0.653 (0.532-0.773) |
| PC aa C34:2 | 185.030 | 0.425 | 0.825 | 0.526 (0.392-0.659) |
| PC aa C34:3 | 3.847 | 0.175 | 0.950 | 0.471 (0.339-0.604) |
| PC aa C34:4 | 0.721 | 0.700 | 0.825 | 0.773 (0.664-0.882) |
| PC aa C36:0 | 4.828 | 0.775 | 0.925 | 0.833 (0.731-0.934) |
| PC aa C36:1 | 30.246 | 0.525 | 0.750 | 0.606 (0.477-0.736) |
| PC aa C36:2 | 108.291 | 0.525 | 0.925 | 0.681 (0.554-0.808) |
| PC aa C36:3 | 70.382 | 0.500 | 0.825 | 0.643 (0.517-0.768) |
| PC aa C36:4 | 113.533 | 0.525 | 0.700 | 0.530 (0.398-0.662) |
| PC aa C36:5 | 7.762 | 0.700 | 0.600 | 0.651 (0.530-0.772) |
| PC aa C36:6 | 0.384 | 0.650 | 0.825 | 0.706 (0.584-0.828) |
| PC aa C38:0 | 2.026 | 0.725 | 0.850 | 0.761 (0.652-0.871) |
| PC aa C38:3 | 27.838 | 0.675 | 0.850 | 0.739 (0.624-0.855) |
| PC aa C38:4 | 58.990 | 0.650 | 0.675 | 0.654 (0.530-0.778) |
| PC aa C38:5 | 27.128 | 0.775 | 0.625 | 0.674 (0.552-0.796) |
| PC aa C38:6 | 41.013 | 0.575 | 0.700 | 0.612 (0.484-0.740) |
| PC aa C40:1 | 0.500 | 0.652 | 0.722 | 0.640 (0.494-0.786) |
| PC aa C40:2 | 0.479 | 0.625 | 0.725 | 0.645 (0.518-0.772) |
| PC aa C40:3 | 0.418 | 0.450 | 0.875 | 0.629 (0.502-0.756) |
| PC aa C40:4 | 2.069 | 0.600 | 0.800 | 0.708 (0.589-0.827) |
| PC aa C40:5 | 4.958 | 0.600 | 0.750 | 0.670 (0.548-0.792) |
| PC aa C40:6 | 10.853 | 0.425 | 0.875 | 0.650 (0.526-0.774) |
| PC aa C42:0 | 0.389 | 0.600 | 0.700 | 0.666 (0.547-0.785) |
| PC aa C42:1 | 0.238 | 0.775 | 0.725 | 0.774 (0.668-0.880) |
| PC aa C42:2 | 0.194 | 0.650 | 0.875 | 0.761 (0.650-0.872) |
| PC aa C42:4 | 0.220 | 0.700 | 0.575 | 0.634 (0.510-0.759) |
| PC aa C42:5 | 0.231 | 0.600 | 0.750 | 0.633 (0.505-0.761) |
| PC aa C42:6 | 0.326 | 0.675 | 0.800 | 0.724 (0.607-0.842) |
| PC ae C30:0 | 0.430 | 0.590 | 0.846 | 0.698 (0.576-0.819) |
| PC ae C30:1 | 0.403 | 0.600 | 0.775 | 0.680 (0.558-0.802) |
| PC ae C30:2 | 0.272 | 0.775 | 0.744 | 0.751 (0.639-0.862) |
| PC ae C32:1 | 1.572 | 0.550 | 0.775 | 0.615 (0.485-0.745) |
| PC ae C32:2 | 0.455 | 0.800 | 0.675 | 0.750 (0.636-0.864) |
| PC ae C34:0 | 0.849 | 0.400 | 0.875 | 0.534 (0.399-0.669) |
| PC ae C34:1 | 9.803 | 0.300 | 1.000 | 0.554 (0.421-0.686) |
| PC ae C34:2 | 4.910 | 0.625 | 0.875 | 0.741 (0.627-0.855) |
| PC ae C34:3 | 3.166 | 0.775 | 0.850 | 0.833 (0.735-0.930) |
| PC ae C36:0 | 1.392 | 0.675 | 0.650 | 0.624 (0.494-0.755) |
| PC ae C36:1 | 6.780 | 0.400 | 0.900 | 0.586 (0.453-0.718) |
| PC ae C36:2 | 5.593 | 0.425 | 0.900 | 0.601 (0.470-0.732) |
| PC ae C36:3 | 4.194 | 0.825 | 0.750 | 0.824 (0.730-0.918) |
| PC ae C36:4 | 8.805 | 0.725 | 0.825 | 0.809 (0.713-0.905) |
| PC ae C36:5 | 6.351 | 0.650 | 0.800 | 0.781 (0.680-0.881) |
| PC ae C38:0 | 0.940 | 0.475 | 0.950 | 0.665 (0.539-0.791) |
| PC ae C38:1 | 3.557 | 0.800 | 0.875 | 0.886 (0.810-0.962) |
| PC ae C38:2 | 3.220 | 0.875 | 0.675 | 0.814 (0.714-0.914) |
| PC ae C38:3 | 4.126 | 0.675 | 0.800 | 0.759 (0.649-0.868) |
| PC ae C38:4 | 7.201 | 0.625 | 0.850 | 0.719 (0.600-0.837) |
| PC ae C38:5 | 11.633 | 0.775 | 0.575 | 0.722 (0.609-0.835) |
| PC ae C38:6 | 3.542 | 0.675 | 0.950 | 0.829 (0.736-0.923) |
| PC ae C40:1 | 1.019 | 0.725 | 0.925 | 0.875 (0.798-0.952) |
| PC ae C40:2 | 1.207 | 0.500 | 0.900 | 0.684 (0.562-0.806) |
| PC ae C40:3 | 1.376 | 0.700 | 0.875 | 0.814 (0.713-0.915) |
| PC ae C40:4 | 1.890 | 0.750 | 0.775 | 0.789 (0.687-0.891) |
| PC ae C40:5 | 2.741 | 0.650 | 0.825 | 0.764 (0.657-0.870) |
| PC ae C40:6 | 2.646 | 0.700 | 0.825 | 0.768 (0.659-0.876) |
| PC ae C42:1 | 0.302 | 0.650 | 0.700 | 0.639 (0.514-0.764) |
| PC ae C42:2 | 0.396 | 0.725 | 0.825 | 0.780 (0.677-0.883) |
| PC ae C42:3 | 0.617 | 0.925 | 0.625 | 0.849 (0.765-0.933) |
| PC ae C42:4 | 0.692 | 0.825 | 0.725 | 0.784 (0.682-0.886) |
| PC ae C42:5 | 1.461 | 0.625 | 0.775 | 0.704 (0.588-0.821) |
| PC ae C44:3 | 0.120 | 0.615 | 0.725 | 0.661 (0.538-0.784) |
| PC ae C44:4 | 0.266 | 0.600 | 0.875 | 0.781 (0.681-0.881) |
| PC ae C44:5 | 1.162 | 0.500 | 0.825 | 0.646 (0.524-0.769) |
| PC ae C44:6 | 0.701 | 0.625 | 0.825 | 0.706 (0.588-0.824) |
| SM (OH) C14:1 | 3.179 | 0.350 | 0.875 | 0.604 (0.479-0.729) |
| SM (OH) C16:1 | 1.991 | 0.525 | 0.575 | 0.463 (0.333-0.592) |
| SM (OH) C22:1 | 7.323 | 0.879 | 0.950 | 0.951 (0.904-0.997) |
| SM (OH) C22:2 | 6.267 | 0.658 | 0.825 | 0.772 (0.669-0.876) |
| SM C16:0 | 66.069 | 0.575 | 0.675 | 0.603 (0.477-0.729) |
| SM C16:1 | 8.765 | 0.525 | 0.750 | 0.639 (0.515-0.763) |
| SM C18:0 | 16.010 | 0.700 | 0.475 | 0.526 (0.396-0.656) |
| SM C18:1 | 5.396 | 0.300 | 0.925 | 0.548 (0.419-0.676) |
| SM C24:0 | 19.173 | 0.875 | 0.800 | 0.890 (0.817-0.963) |
| SM C24:1 | 18.581 | 0.800 | 0.750 | 0.760 (0.614-0.906) |
| H1 | 3795.314 | 0.825 | 0.825 | 0.839 (0.744-0.933) |
| Progesteron | 0.138 | 0.975 | 1.000 | 0.999 (0.996-1.000) |
| 5-α-Cholestane | 0.965 | 0.850 | 0.775 | 0.895 (0.828-0.962) |
| Chol_Epoxide | 1.112 | 1.000 | 1.000 | 1.000 (1.000-1.000) |
| 1,2dilinoleoyl_PC | 12.817 | 0.850 | 1.000 | 0.939 (0.879-0.999) |
| 1,2dioleoyl_GLP_Na2 | 2.916 | 1.000 | 1.000 | 1.000 (1.000-1.000) |
| D-sphingosine | 0.185 | 0.925 | 0.975 | 0.942 (0.877-1.000) |
| C16-CAR2 | 0.063 | 0.750 | 0.950 | 0.851 (0.747-0.956) |
| Oleoyl-CAR | 0.954 | 1.000 | 1.000 | 1.000 (1.000-1.000) |
| Lanosterol | 80.391 | 1.000 | 1.000 | 1.000 (1.000-1.000) |
| CER_893_1 | 0.747 | 0.625 | 0.775 | 0.717 (0.599-0.835) |
| 1-palmitoyl-sn-glycero-3PC | 240.785 | 1.000 | 0.875 | 0.969 (0.935-1.000) |
| BSitosterol | 3.874 | 0.525 | 0.925 | 0.690 (0.563-0.817) |
| glyceryltrioleate1 | 58.270 | 0.800 | 0.425 | 0.593 (0.467-0.719) |
| 1,2dioleoyl_PE | 0.529 | 1.000 | 0.775 | 0.931 (0.871-0.991) |
| cis-vaccenic_acid | 70.286 | 0.600 | 0.825 | 0.742 (0.632-0.852) |
| ArachidicAcid | 3.049 | 0.925 | 0.975 | 0.981 (0.959-1.000) |
| erucic acid | 1.467 | 1.000 | 1.000 | 1.000 (1.000-1.000) |
| StearicAcid | 113.039 | 0.975 | 1.000 | 0.998 (0.994-1.000) |
| PalmiticAcid | 134.376 | 1.000 | 1.000 | 1.000 (1.000-1.000) |
| LinoleicAcid | 44.805 | 0.425 | 0.875 | 0.641 (0.514-0.768) |
| DocosahexaenoicAcid | 4.732 | 0.775 | 0.850 | 0.883 (0.808-0.957) |
| PalmitoleicAcid | 19.150 | 0.850 | 0.750 | 0.859 (0.778-0.940) |
| BehenicAcid | 1.837 | 1.000 | 0.975 | 0.999 (0.998-1.000) |
| MyristicAcid | 24.198 | 0.675 | 0.600 | 0.587 (0.456-0.717) |
| LinolenicAcid | 3.845 | 0.650 | 0.775 | 0.730 (0.615-0.845) |
| OleicAcid | 90.718 | 0.825 | 0.425 | 0.642 (0.519-0.765) |
| MyristoleicAcid | 1.434 | 0.725 | 0.625 | 0.652 (0.530-0.774) |
| LignocericAcid | 1.800 | 1.000 | 1.000 | 1.000 (1.000-1.000) |
| MargaricAcid | 4.241 | 1.000 | 0.775 | 0.947 (0.901-0.992) |
| oleanolic acid | 2.693 | 1.000 | 1.000 | 1.000 (1.000-1.000) |
| tripentadecanoate TG15 | 4.748 | 0.975 | 0.975 | 0.988 (0.966-1.000) |
| Glyceryltrilinoleate1 | 11.422 | 1.000 | 0.875 | 0.979 (0.954-1.000) |
| glyceryltripalmitoleate1 | 0.979 | 0.400 | 0.850 | 0.584 (0.456-0.712) |
| 1linoleoyl-rac-GL | 4.269 | 0.825 | 1.000 | 0.837 (0.725-0.949) |
| 1oleoyl_rac_GL | 12.530 | 1.000 | 1.000 | 1.000 (1.000-1.000) |
| 1monopalmitoleoyl-rac-GL1 | 11.897 | 0.975 | 1.000 | 0.997 (0.990-1.000) |
| desmosterol1 | 0.530 | 0.325 | 0.975 | 0.602 (0.474-0.730) |
| CHOLESTEROL2 | 318.718 | 0.694 | 0.875 | 0.826 (0.731-0.922) |
| CA | 0.413 | 0.700 | 1.000 | 0.779 (0.661-0.897) |
| CDCA | 0.208 | 0.475 | 1.000 | 0.683 (0.559-0.808) |
| DCA | 0.071 | 0.600 | 0.925 | 0.747 (0.629-0.865) |
| UDCA | 0.046 | 0.525 | 0.900 | 0.752 (0.643-0.861) |
| LCA | 0.178 | 0.650 | 0.718 | 0.678 (0.557-0.799) |
| G-CA | 0.617 | 0.600 | 0.950 | 0.706 (0.579-0.833) |
| G-CDCA | 0.598 | 0.900 | 0.825 | 0.930 (0.879-0.981) |
| G-DCA | 0.328 | 0.450 | 0.850 | 0.637 (0.512-0.762) |
| G-UDCA | 0.090 | 0.775 | 0.425 | 0.587 (0.461-0.713) |
| G-LCA | 0.031 | 1.000 | 0.667 | 0.812 (0.711-0.912) |
| T-CA | 0.210 | 0.550 | 0.975 | 0.726 (0.602-0.849) |
| T-CDCA | 0.257 | 1.000 | 0.950 | 0.984 (0.961-1.000) |
| T-DCA | 0.313 | 0.525 | 1.000 | 0.678 (0.547-0.808) |
| T-UDCA | 0.037 | 0.541 | 1.000 | 0.568 (0.411-0.724) |
| T-LCA | 0.237 | 1.000 | 1.000 | 1.000 (1.000-1.000) |
